# Soil Bacterial Community Shifts after Chitin Enrichment: An Integrative Metagenomic Approach

**DOI:** 10.1371/journal.pone.0079699

**Published:** 2013-11-20

**Authors:** Samuel Jacquiod, Laure Franqueville, Sébastien Cécillon, Timothy M. Vogel, Pascal Simonet

**Affiliations:** 1 Environmental Microbial Genomics Group, Ecole Centrale de Lyon, Laboratoire Ampère UMR5005 CNRS, Ecully, France; 2 Microbial Molecular Ecology Group, Section of Microbiology, København Universitat, København, Denmark; Tel Aviv University, Israel

## Abstract

Chitin is the second most produced biopolymer on Earth after cellulose. Chitin degrading enzymes are promising but untapped sources for developing novel industrial biocatalysts. Hidden amongst uncultivated micro-organisms, new bacterial enzymes can be discovered and exploited by metagenomic approaches through extensive cloning and screening. Enrichment is also a well-known strategy, as it allows selection of organisms adapted to feed on a specific compound. In this study, we investigated how the soil bacterial community responded to chitin enrichment in a microcosm experiment. An integrative metagenomic approach coupling phylochips and high throughput shotgun pyrosequencing was established in order to assess the taxonomical and functional changes in the soil bacterial community. Results indicate that chitin enrichment leads to an increase of *Actinobacteria, γ-proteobacteria* and *β-proteobacteria* suggesting specific selection of chitin degrading bacteria belonging to these classes. Part of enriched bacterial genera were not yet reported to be involved in chitin degradation, like the members from the *Micrococcineae* sub-order (*Actinobacteria*). An increase of the observed bacterial diversity was noticed, with detection of specific genera only in chitin treated conditions. The relative proportion of metagenomic sequences related to chitin degradation was significantly increased, even if it represents only a tiny fraction of the sequence diversity found in a soil metagenome.

## Introduction

Chitin is an homopolymer of β-1,4-linked N-acetylglucosamine (N-acetyl-D-glucose-2-amine, NAG) with a critical biological role in both terrestrial and aquatic ecosystems as a major constituent of fungi and plant cell walls, as well as insect, krill and shellfish exoskeletons [1]. Chitin is the second most abundant biopolymer in nature after cellulose, with an estimated natural production of 10^10^ tons per year [2], [3]. The degradation of chitin releases substantial amounts of carbon and nitrogen nutrients in terrestrial [4] [5] [6], aquatic [2] [7] [8] and sediment ecosystems [9]. However, its crystallized conformation and heterogenic chemical composition make chitin particularly recalcitrant to degradation [10]. Total mineralization is carried out only by a highly specialized microflora through specific microbiological enzymatic processes. Up to now, efficient bacterial chitin degraders were isolated through culture, including representatives from *Actinobacteria* (e.g. *Streptomyces sp*.) [11], *β-proteobacteria* (e.g. *Burkholderia sp.*) [12] and *γ-proteobacteria* (e.g. *Xanthomonas sp.*) [13]. However, uncultured and unknown bacteria might also be involved in the degradation process as active key players (e.g. cooperating degraders), or passive players (e.g. opportunist cheaters), through establishment of local biofilm structures on chitin fibers [14], [15]. Apart from their fundamental role in ecosystems functioning, chitin degraders and their enzymes received a particular attention during the last decade for numerous applications. Chitin degraders are candidates for *in situ* application as biocontrol agents of soil born plant-pathogenic fungi [11], while shellfish wastes can be treated in the frame of enzymatic industrial processes, involving new and efficient chitinases characterized from environmental microbial communities [16], [17].

All known enzymes involved in chitin degradation are classified in the CAZy database (Carbohydrate Active Enzymes, http://www.CAZy.org/, Accessed 2013 October 09) [18]. Chitinases (EC 3.2.1.14) belong to glycosyl hydrolases families GH18 and GH19 based on amino acid sequence similarity [19], [20]. Enzymes harboring carbohydrate binding modules (CAZy Auxiliary Activity enzymes AAs, former CMB33) also display lytic activities toward chitin and are known to act as “facilitating enzymes” acting on crystalline chitin (e.g. monooxygenases), and helping in releasing fibers for further degradation by chitinases [21]. Another chitin degradation pathway relies on chitin deacetylases (EC 3.5.1.41), belonging to the carbohydrate esterase family CE4 [22], and chitosanases (EC 3.2.1.132), belonging to families GH46 and GH75. Chitinases are also classified based on their depolymerization activity, including endochitinases (EC 3.2.1.14), which randomly cleave chitin molecules and exochitinases, such as β-(1,4)-N-acetylglucosaminidases (EC 3.2.1.30) and 1,4-β-chitobiosidases (EC 3.2.1.29,) which progressively degrade chitin molecules from non-reduced ends [23], [24].

Environmental bacteria represent an untapped reservoir of enzymatic diversity considering that metagenomic approaches have not yet been extensively applied for detection of new chitin degrading enzymes (e.g. ocean water [25], lakes [2], soils [11], [26] and sediments [9], [27]). This indicates the difficulty of detecting new genes of interest even by culture independent approaches. The complexity of environmental metagenomes, related to the high level of diversity, considerably limits attempts to exploit hidden enzymatic resources. The number of clones in metagenomic libraries must be scaled up significantly in order to increase chances of detecting rare genes. This effort also requires specific robots and facilities, which are often not accessible for most research groups. An alternative strategy would be to increase the proportion of targeted genes in the bacterial community to facilitate their detection. Addition of colloidal chitin to active environmental samples is commonly applied in soil microbiology studies [28], [29]. This approach is expected to significantly increase the fitness of chitin degrading bacteria, and consequently increase the proportion of genes related to chitin degradation in the subsequently extracted metagenomic DNA. As an indication, cultured chitin degrading bacteria are known to harbor, on average, 5 chitinolytic genes [25]. In previous studies, several abiotic parameters involved in chitin degradation were tested, such as moisture [30], pH [26], temperature and soil types [31], but the effect of chitin concentration has never been investigated. In this work, we tested the impact of chitin concentration on the taxonomical and functional structure of soil bacterial community through an enrichment approach in microcosms. The well characterized soil from the Park Grass Experiment (Rothamsted Research Station, UK) was used to perform this study. Metagenomic DNA was extracted from soil samples incubated for 35 days in microcosms with two different concentration of colloidal shrimp chitin: 1×(low treatment, 2 mg/g of soil), 10×(high treatment, 20 mg/g of soil) and a control 0×. Analysis of the bacterial community was performed through the combinatory use of complementary techniques including enzymatic assays, RISA (Ribosomic Intergenic Spacer Analysis), 16S rRNA qPCR, phylochips and shotgun pyrosequencing for the most promising samples. The metagenomic reference database generated from the same soil in the frame of the Terragenome consortium was used as a control in order to reinforce the comparative analysis [32], [33]. A flowchart summary presenting the experimental design of this study is presented in Supplemental Data (File S1).

## Materials and Methods

### Soil Sampling

Fresh soil cores were collected from Park Grass (lat 51.481481°N, long 0.222231°E), Rothamsted, England (Sampling permission issued by the Rothamsted Research Center, see http://www.rothamsted.ac.uk/for further information, Accessed 2013 October 09). The Park Grass from Rothamsted is an internationally recognized resource and is selected to be a reference for soil metagenomic studies [32]. The soil from the ParkGrass experiment was monitored over more than 150 years for field-based experiments in order to examine the effects of fertilizers on crops. Soil samples and meteorological records were continuously accumulated since the beginning of experiments (1856), resulting in constitution of an important database on this site. The soil samples from this study are coming from the control part of the site, which has received no specific treatment since then. Soil is classified as chromic luvisol based on FAO guidelines [34] and is a silty clay loam overlying clay with flints with a pH of 5.2 (measured in H_2_O). Park Grass covers 249 m^2^ (13.28 by 18.75 m), and the sampling strategies consisted of harvesting randomized soil samples in the plot. The soil cores were around 6 cm diameters for 20 cm depth, and were collected into plastic bags and rapidly transferred at Ecole Centrale de Lyon (France). Soil samples were sieved at 2 mm, pooled, and directly used to run microcosm experiments. All tools and materials used were washed and cleaned with 70% ethanol solution.

### Microcosm’s Settings, Chitin Enrichment and Sampling Strategy

Three enrichment conditions corresponding to two chitin concentrations and a control were designed. Each condition was set in three replicates consisting in glass bottles containing 50 g of sieved soil, and capped with cheese cloth to allow gas exchanges. Colloidal chitin was prepared from shrimp shells (C7170, Sigma-Aldrich, Germany) as described by Inglis and Kawchuk [28]. 15 grams of shrimp shells were dissolved in 50 ml HCl (Roth Sochiel, 37%) and added to 300 ml of sterile deionized water during 4 hours with magnet bar mixing. The solution was neutralized at pH 7.00 with NaOH 0.1 M, and colloidal chitin was recovered after centrifugation (10 mins, 10′000 g). A washing step was performed with 200 mM phosphate buffer pH 7.00, followed by a second centrifugation (10 mins, 10′000 g). Colloidal chitin was chosen as it is known to be more readily degraded by microorganisms [35]. For the lowest chitin concentration, 0.1 g of colloidal chitin was added with 5 ml water and mixed with soil for a final concentration of 2 mg/g^−1^ soil (chitin 1×). For the highest concentration, 1 g of colloidal chitin was added in 5 ml water, corresponding to 20 mg/g^−1^ soil (chitin 10×). This represents 0.49 mM and 4.9 mM of chitin per bottle, respectively (Chitin molecular weight = 203.1925 g/mol^−1^). Control bottles were amended with 5 ml of water as well (control 0×). Soil water saturation was around 70% in all conditions. Microcosms were incubated under green-house conditions with a constant temperature of 24°C and 65% relative humidity. One gram of soil was sampled for each replicate bottle and for each condition respectively after 0, 3, 6, 10, 20, and 35 days of enrichment. Soil samples were stored at −20°C until the end of the experiment.

### Chitin Degradation and Chitinase Assays

200 mg of soil were collected separately from the three microcosm replicates and mixed with 0.4 ml of Dulbecco’s phosphate buffer saline pH 8.00. Samples were mixed in a vortex for 2 mins at maximum speed (Vortex Genie 2, SCIENTIFIC INDUSTRIES). The mixture was clarified by centrifugation at 13′000 g and 500 µl of supernatant was transferred in a clean collection tube and stored on ice. Enzymatic assays were performed on 10 µl of supernatant with a fluorometric chitinase assay kit following manufacturer’s instructions (CS1030, Sigma-Aldrich, Germany, Lifesciences). Several synthetic substrates are provided for detecting three enzymatic activities related to chitin degradation. In this study, we only considered the chitinase activity detected with 4-methylumbelliferyl β-D-N′,N″-triacetylchitotriose, a substrate suitable for detection of both exo- and endochitinase activity based on manufacturer’s instructions, and previous reported activity results [25], [36]. After degradation, substrate releases 4-methylumbelliferone (4 MU), a fluorescent compound emitting at 450 nm when excited at 360 nm. Fluorescence was measured after 45 mins incubation at 37°C on a microplate reader (Infinite 1000, TECAN) and activities were stated with a 4 MU standard curve. Enzymatic activities were normalized based on the incubation time, and expressed in chitinase unity detected per gram of soil (1 U = 1 µmole 4 MU released per minute). The amount of colloidal chitin degraded (M) over the incubation time for each condition can be calculated with a moving average as follow:
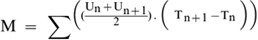



In this calculus, “*Tn*” is representing a given time point in minutes since the starting point of the enrichment kinetic. “*Tn+1–Tn*” is giving the amount of incubation time between two sampling points. “*Un*” is representing the chitinase activity measured in µmole 4 MU/min^−1^ for a given time point. “*1/2(Un+Un+1)*” is giving the moving average of chitinase activity between two given time points.

### Metagenomic DNA Extraction

Metagenomic DNA was extracted from each replicate using an adapted protocol from Griffith and co-authors [37]. 500 mg of soil were placed in FastPrep-24 lysing matrix tube (MP Bio1O1, MP Biomedicals). Cell lysis was performed in a FastPrep-24 beadbeater (MP Biomedicals), at 5.5 speed during 30 s with 600 µl phenol:chloroform:isoamyl alcohol (Roth, 24∶24∶1) and 600 µl extraction buffer (300 µl phosphate buffer pH 8.00, 300 µl 10% acetyltrimethylammonium bromide/700 mM NaCl). Supernatant aqueous phase was recovered after centrifugation (5 mins, 16′000 g, 4°C). A second treatment was performed on the recovered aqueous phase with 500 µl chloroform isoamyl alcohol, followed by short vortex mix, and centrifugation (5 mins, 16′000 g, 4°C). Total DNA was precipitated over night at 4°C with 2 volumes of absolute ethanol and 1/10 volume of NaCl 5 M. Metagenomic DNA was purified on silica column according to manufacturer instructions (Illustra™ GFX™ PCR DNA and Gel Band Purification Kit, GE Healthcare). DNA was quantified by fluorometric assays (Qubit fluorometer, Invitrogen, Life technologies). Extraction reproducibility was evaluated over the 3 replicates of each condition through RISA (Ribosomic Intergenic Spacer Analysis), a bacterial community fingerprinting technique [38]. This part of the work is presented in Supplemental Data (File S2).

### 16S rRNA qPCR

Partial 16S rRNA genes were directly amplified from diluted metagenomic DNA solutions obtained for each of the 3 replicates per condition. Eubacterial primers Eub338 5′ -ACTCCTACGGGAGGCAGCAG-3′ (forward) and Eub 518 5′-ATTACCGCGGCTGCTGG-3′ (reverse) were used [39]. 2 µl of diluted metagenomic DNA (≈15 ng) were mixed with 0.4 µl of reverse and forward primers (5 µM), 10 µl of qPCR buffer Sensimix® (Bioline), 7.2 µl of distilled sterile water. qPCR assays were performed in a RotorGene RG-6000 (Corbett Research, QIAGEN) with the following conditions: 10 mins at 95°C, followed by 35 cycles at 95°C for 20 s and annealing at 53°C for 20 s, and elongation at 72°C for 20 s. The standard range was performed using purified 16S rRNA products amplified with the same set of primers from the original soil metagenomic DNA. A standard curve ranging from 10^4^ to 10^8^ molecular copies was used to quantify the 16S rRNA gene in samples (Efficiency = 0.99, R^2^ = 0.999). Results were analyzed with the manufacturer’s software (Rotor-Gene 6000 Series Software 1.7). Because the 16S rRNA gene copy number is strongly varying depending on bacteria species [40], the quantification results were normalized based on DNA extraction yields per gram of soil.

### Phylochip Analysis

The microarray format used in this study was from Agilent Sureprint Technologies, consisting in 8 blocks of 15′000 spots each, designed on a standard glass slide (25 mm×75 mm). Each spot holds a 20-mer oligonucleotide probe synthesized *in situ*. Each oligonucleotide probe occurred at least in triplicate within each block. All blocks were identical. This format allows hybridization of eight samples per slide at the time. Probes target the 16S rRNA gene covering a wide part of the *Bacteria* and *Archaea* phylogenic tree. Probes were designed with the ARB software package and the PhylArray software [41]. The 20-mer probes have a final melting temperature of 65°C ±5°C and less than 1.5 weighted mismatches. Our design includes oligonucleotide probes at different taxonomic levels. This microarray covers over 400 genera and 10000 OTUs (“species” or “hits”).

The reproducibility of the taxonomical profiles between bottle replicates was tested and validated with preliminary phylochips, as described in Supplemental Data (File S3). The bacterial 16S rRNA gene was amplified from pooled metagenomic DNA, corresponding to microcosm replicates from the original soil 0×0 before incubation, and chitin enriched samples from day20∶0×20, 1×20 and 10×20. The PCR reaction was performed using universal primer *pA* 5′-TAATACGACTCACTATAGAGAGTTTGATCCTGGCTCAG-3′ and *pH-T7* 5′-AAGGAGGTGATCCAGCCGCA-3′ [42]. 2 µl of diluted metagenomic DNA (≈15 ng) was mixed with 1.5 µl of reverse and forward primers (10 µM), 45 µl of distilled sterile water, and 1 µl Taq Polymerase (Invitrogen). PCR was conducted at 94°C for 4 mins and then with 35 cycles of 94°C for 45 s, annealing at 55°C for 45 s, and elongation at 68°C for 95 s, followed by 68°C for 5 mins. Amplified PCR products were loaded on a 1% agarose gel, and after electrophoresis the desired 1500 pb band was extracted and purified (Illustra™ GFX™ PCR DNA and Gel Band Purification Kit, GE Healthcare). Purified PCR products were then transcribed into RNA using T7 rRNA polymerase (Invitrogen) with the incorporation of labeled Cy5-UTP (GE Healthcare). Cy5 is a fluorescent dye, emitting at 670 nm after excitation at 650 nm. rRNA purification was performed with the Qiagen RNeasy minikit based on manufacturer’s instructions. Chemical rRNA fragmentation was achieved by the addition of 1.14 µl of Tris-Cl (1 mM) and 4.57 µl of ZnSO_4_ (100 mM) to 40 µl of labeled RNA sample and incubation for 30 mins at 60°C.The fragmented and labeled rRNA was hybridized overnight on the phylochips at 60°C, and washed with the buffer supplied by the manufacturer.

### Microarray Scanning and Data Processing

An Innoscan 700 scanner (Carbonne, France) was used for scanning microarray slides based on manufacturer’s instructions. Raw hybridization fluorescence signal for each spot was determined based on the signal-to-noise ratio (SNR), which was calculated by using the following formula: SNR = (signal intensity – background)/background standard deviation. Total probe fluorescence signal, including negative controls, was transformed by calculating the signal in log_2_. Since at least three replicates exist for all oligonucleotide probes, outliers were eliminated when any individual spot was greater than 3 standard deviations from the average of all replicates. Analysis of variance (ANOVA) was used to evaluate positive probes. Since probes have different phylogenetic depths, the genera described here were those for which all relevant probes were positive. While all probes could not be independently verified, many of them were validated by the application of DNA from a single bacterium [43]. Since environmental bacteria are holding different16S rRNA gene copy numbers [40], hybridization signal intensity is known to be extremely biased when interpreting quantitative aspects of phylochip data. As a consequence, the interpretation will be done only through a relative comparative analysis between the samples, as the same bias will occur in all tested conditions. Application of the phylochip design, and its related data processing, was already validated in a previous study [44].

### Pyrosequencing and Data Analysis

Up to 10 µg of metagenomic DNA corresponding to the enrichment time day 20 were extracted and purified as described in section “*Metagenomic DNA extraction*” for each condition and replicate. Based on preliminary analysis (File S2 and File S3), only two replicates were sent for further pyrosequencing investigation. Pyrosequencing replicates are indicated as: 0×20a, 0×20b, 1×20a, 1×20b, 10×20a and 10×20b. Metagenomic DNA was sequenced with the 454 Titanium pyrosequencing technology (Roche) at the Genoscope Sequencing Center (Paris, France). Raw data were cleaned from artificial duplicates using the CD-HIT-454 software [45]. On average, each metagenome yielded 559671 reads (+/−6.3×10^4^), with an average read size of 392 bp (+/−32) and an average GC content of 63% (+/−0.5). Metagenomic datasets were analyzed through read annotation using the MG-RAST platform for both functional and taxonomical annotations [46]. The taxonomical affiliation of reads was done against the M5NR public database. The functional affiliation of reads was done against the SEED database [47], which is based on a hierarchical classification of reads into subsystems related to specific functions. The input parameters for both taxonomical and functional annotation were: 60% minimal identity, 30 bp minimal alignment length, and 1.E-05 e-value threshold. Statistical analyses were performed with Rgui [48] and STAMP software [49]. Two pyrosequencing datasets corresponding to the original soil at day 0∶0×0a and 0×0b were used for comparison [33] (Table 1). The reference metagenomic database generated from the Park Grass soil was used as a control to compare the different conditions, and validate the changes observed after microcosm incubation and chitin treatment [33]. As the repetition number is low (n = 2 for 0×20, 1×20 and 10×20), a multigroup ANOVA was performed with a Welch’s post-hoc correction test as an indication for detecting the main differences between datasets [49]. Only relevant differences (p-value<0.05) were considered during the analysis.

**Table 1:** Description of all the metagenomes used in this study.

Name	Extraction protocol	Sampling date	Depth (cm)	Reference
Roth-F2a	Indirect MP Bio1O1	February 2009	0–21	
Roth-Fb2	Indirect MP Bio1O1	February 2009	0–21	
Roth-F3	Indirect lysis in plug	February 2009	0–10	
Roth-F4	Indirect DNA Tissue	February 2009	0–10	
Roth-F5	Indirect Gram positive	February 2009	0–10	
Roth-F6	Indirect lysis in plug	February 2009	0–10	
Roth-J4	Indirect DNA Tissue	July 2009	0–10	Delmont et al. 2012
Roth-F1	Direct MP Bio1O1	February 2009	0–21	
Roth-J1	Direct MP Bio1O1	July 2009	0–21	
Roth-J2	Direct MP Bio1O1	July 2010	0–21	
Roth-J7	Direct MoBio	July 2009	0–21	
0×0a	Direct MP Bio1O1	July 2010	0–21	
0×0b	Direct MP Bio1O1	July 2010	0–21	
0×20a	Direct MP Bio1O1	July 2010	0–21	
0×20b	Direct MP Bio1O1	July 2010	0–21	
1×20a	Direct MP Bio1O1	July 2010	0–21	This study
1×20b	Direct MP Bio1O1	July 2010	0–21	
10×20a	Direct MP Bio1O1	July 2010	0–21	
10×20b	Direct MP Bio1O1	July 2010	0–21	

DNA extraction protocols are referring to different strategies including a wide range of approach to extract and lyse cells. Direct approaches are relying on *in situ* cell lysis within the soil sample, while indirect approaches are relying on bacterial cell separation from the sample matrix before performing the lysis.

### Metagenomes Deposition

The 6 soil metagenomes generated in this study are publically available on the MG-RAST public database (http//http://metagenomics.anl.gov/, Accessed 2013 October 09) under the following access numbers (MG-RAST ID): 4537190.3 and 4537191.3 for untreated controls “Roth-Chitin-Control-0×20A(B)”; 4537194.3 and 4537195.3 for low chitin treatment “Roth-Chitin-Low-1×20A(B)”; 4537192.3 and 4537193.3 for high chitin treatment “Roth-Chitin-High-10×20A(B)”.

## Results

### Chitinase Activity

**Figure 1:**
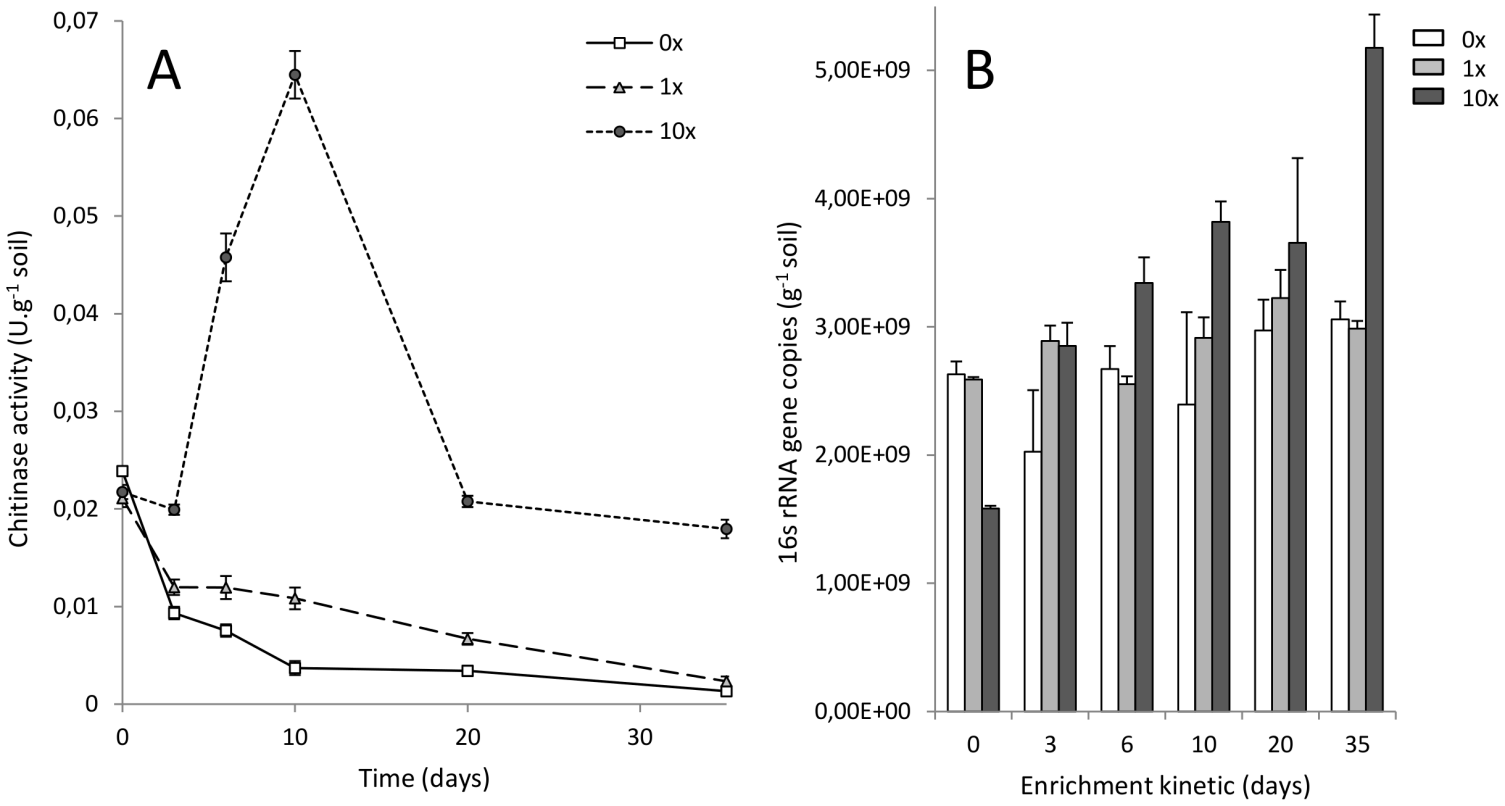
Chitinase activity and 16S-rRNA qPCR quantification during microcosm enrichment. Panel A displays chitinase assays performed with synthetic substrates on soil aqueous extracts. Activity was measured by quantification of the fluorescence release by the 4-Methylumbelliferon (4 MU) after specific cleavage of exo- and endochitinases. Enzyme activity is expressed in chitinase unity detected per gram of soil during incubation time (1 U = 1 µmole 4 MU released per minute). Panel B shows 16S-rRNA gene copies detected by qPCR per gram of soil. Results were normalized with obtained DNA yields and expressed per gram of soil. The enrichment kinetic is represented in days. In both panels, chitin concentration is represented by darker gray nuance: light grey (low chitin dose 1×), dark grey (high chitin dose 10×) and white (control 0×). The error bars are representing the standard deviation observed in the 3 microcosm replicates (SD).

Results presented in Fig. 1, panel A indicate that the main initial impact on chitinase activity was due to soil incubation in microcosm, as shown by the early drop in the control 0×. In spite of higher activity rates, the 1× condition followed the same trend as the control, with a gradual decrease over time except between day 3 and 10 where the activity seemed to stabilize due to low chitin input. The 10× chitin treated microcosms displayed a clear activity peak after 10 days, followed by a rapid activity drop at day 20 to finally reach its initial level. Chitinase activity of 10× samples seems to stabilize between day 20 and 35. Based on activity measurements, it was estimated on average that 0.243 mM of chitin was degraded during incubation time in control bottles. On the other hand, 0.412 and 1.578 mM of chitin were degraded on average in 1× and 10× bottles. After removal of the soil background activity 0×, this represents 0.169 and 1.335 mM of chitin degraded over the enrichment, respectively. Based on the initial chitin inputs (0.49 mM of colloidal chitin for 1×, and 4.9 mM for 10×), 34.37% and 27.13% of chitin was degraded in the 1× and the 10× conditions.

### 16S rRNA qPCR

Quantitative evaluation of 16S rRNA gene copy numbers normalized with DNA extraction yields was used to estimate the impact of the chitin amendment on the bacterial community. The microcosm effect was observed again, as revealed by variations in the control 0×. An initial drop within the first three days was observed in the control, followed by slow recovery between day 6 and 20 days (Fig. 1, panel B). A significant decrease of the 16S rRNA gene copy number was detected after direct addition of high chitin concentration at t = 0, with a noticeable difference in 10× chitin amended microcosm in comparison to the control 0× and 1× samples. As this difference appeared after normalization with DNA extraction yields per gram of soil, we hypothesized that colloidal chitin might interfere during the DNA extraction step, resulting in lower yield recovery in comparison to the control 0× and 1× samples. However, additional experiments would be required to support this observation. Along the kinetic, 1× chitin treatment displayed almost the same trend as the control, except at day 3 where an increase was noticed. Nevertheless, a clear biostimulating effect was observed in the 10× chitin amended microcosms soon after the amendment. A gradual increase during the 35 days was detected for this treatment, except at day 20 as all conditions were more or less at the same level due to wide error-bars at this time point. However, even though a clear increase in the DNA extraction yields (data not shown) and the 16S rRNA gene copy numbers was observed, these observations need to be contrasted, as the 16S rRNA gene copy number can vary from 1 up to 15 copies per genome depending on bacterial species [37].

In reference to these previous analyses, and based on preliminary RISA and phylochips data (File S2 and File S3), only DNA from soil samples incubated for 20 days: 0×20, 1×20 and 10×20, as well as the DNA from non-incubated initial control soil 0×0 were selected for additional analyses using more sensitive approaches including phylochips and shotgun pyrosequencing of the metagenomic DNA.

### Phylochip Results

**Figure 2:**
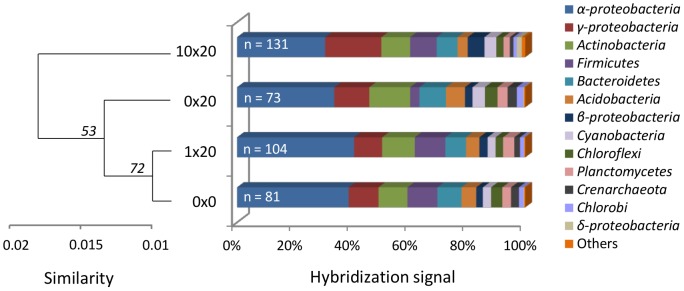
Relative abundance of major prokaryote groups detected on phylochips. Stacked bar chart represents the percentage of total fluorescence signal detected in each group, based on probe hybridizations. The number of identified genera per condition (n) is indicated in white in the chart. Clusters analysis was performed on taxonomical profiles of each sample at the genus level, and exposed to bootstrap simulation (n = 10000). Approximately unbiased bootstrap p-value are expressed in percentage and indicated at each nod. The grouping was done with the Ward method based on variance analysis, and distances are calculated according a correlation algorithm (the complement 1*−r* of Pearson’s *r* correlation). Dendrogram scale is representing the similarity based on the Euclidean distance.

Cluster analysis of phylochip results based on taxonomical profiles at the genus level indicate that the bacterial community from the initial non incubated control soil 0×0 would be more closely related to the 1×20 sample than to the incubated control sample 0×20 (Fig. 2). Only the 10×20 chitin amended sample was clearly separated, indicating significant modifications in its taxonomical profile. This was confirmed at the phylum level, with the specific detection of *Euryarchaeota* and *Verrucomicrobia* in 10×20 (Tab. 2, A). *Euryarchaeota* signals was detected, as *Archaea* probes are present on the microarray, and also because the pH primer used in this study is known to match *Archaea* representatives [50]. In addition, more bacterial genera were detected in chitin amended samples (n = 104 and n = 131 for 1×20 and 10×20, respectively) than in the two control soils (n = 81 and n = 73 for the 0×0 and 0×20, respectively). The 10×20 chitin concentration also revealed the strongest hybridization signal concomitantly with the highest number of genera detected for the *γ-proteobacteria* class, mostly related to the *Xanthomonadales* order. A strong *β-proteobacteria* signal was specifically detected in the 10×20 chitin amended soil, mostly reflecting the abundance of *Burkholderia* genus signal. Even if the signal proportion of *Firmicutes*, *Bacteroidetes* and *Cyanobacteria* at the class level did not seem strongly affected by the treatments, chitin enrichment resulted in unique detection of respectively 16, 3 and 3 genera within these classes (Tab. 2, B) All the genera detected in incubated control 0×20 were also observed in the 3 other conditions, while only 3 genera were only seen in the initial control 0×0. On the other hand, 59 genera were only identified in chitin enriched samples, mostly *γ-proteobacteria* and *Firmicutes* (n = 8 for 1×20, n = 34 for 10×20, and n = 17 for co-occurrence in both 1×20 and 10×20, respectively).

**Table 2:** Counts of genera detected on phylochip for each condition.

	A) Number of genera detected				B) Unique genera detected			
Prokaryote groups	0×0	0×20	1×20	10×20	0×0	1×20	10×20	Chitin treated
*α-proteobacteria* (Class)	22	18	25	25	3	2	3	2
*γ-proteobacteria* (Class)	9	7	17	29			11	9
*Actinobacteria*	10	11	12	15		1	4	
*Firmicutes*	11	9	19	22		4	7	5
*Bacteroidetes*	5	5	5	8			3	
*Acidobacteria*	2	2	2	2				
*β-proteobacteria* (Class)	2	2	4	4		1	1	1
*Cyanobacteria*	8	7	8	11			3	
*Chloroflexi*	2	2	2	2			1	
*Planctomycetes*	2	2	2	2				
*Crenarchaeota*	3	3	3	3				
*Chlorobi*	3	3	3	3				
*δ-proteobacteria* (Class)	1	1	1	2			1	
*Aquificae*	1	1	1	1				
*Euryarchaeota*				1			1	
*Verrucomicrobia*				1			1	
**Total**	**81**	**73**	**104**	**131**	**3**	**8**	**36**	**17**

Panel A) shows the direct counts of genera detected for each prokaryote group on phylochips, while panel B) displays numbers of unique genera observed only under a specific condition. Taxonomy is given at the phylum level, except for *Proteobacteria* which are detailed at the Class level. Total numbers of genera detected per conditions is given in the last row. The last column in Panel B (Chitin treated) is giving the numbers of genera only observed in both chitin treated conditions 1×20 and 10×20.

### Pyrosequencing Analysis

**Figure 3:**
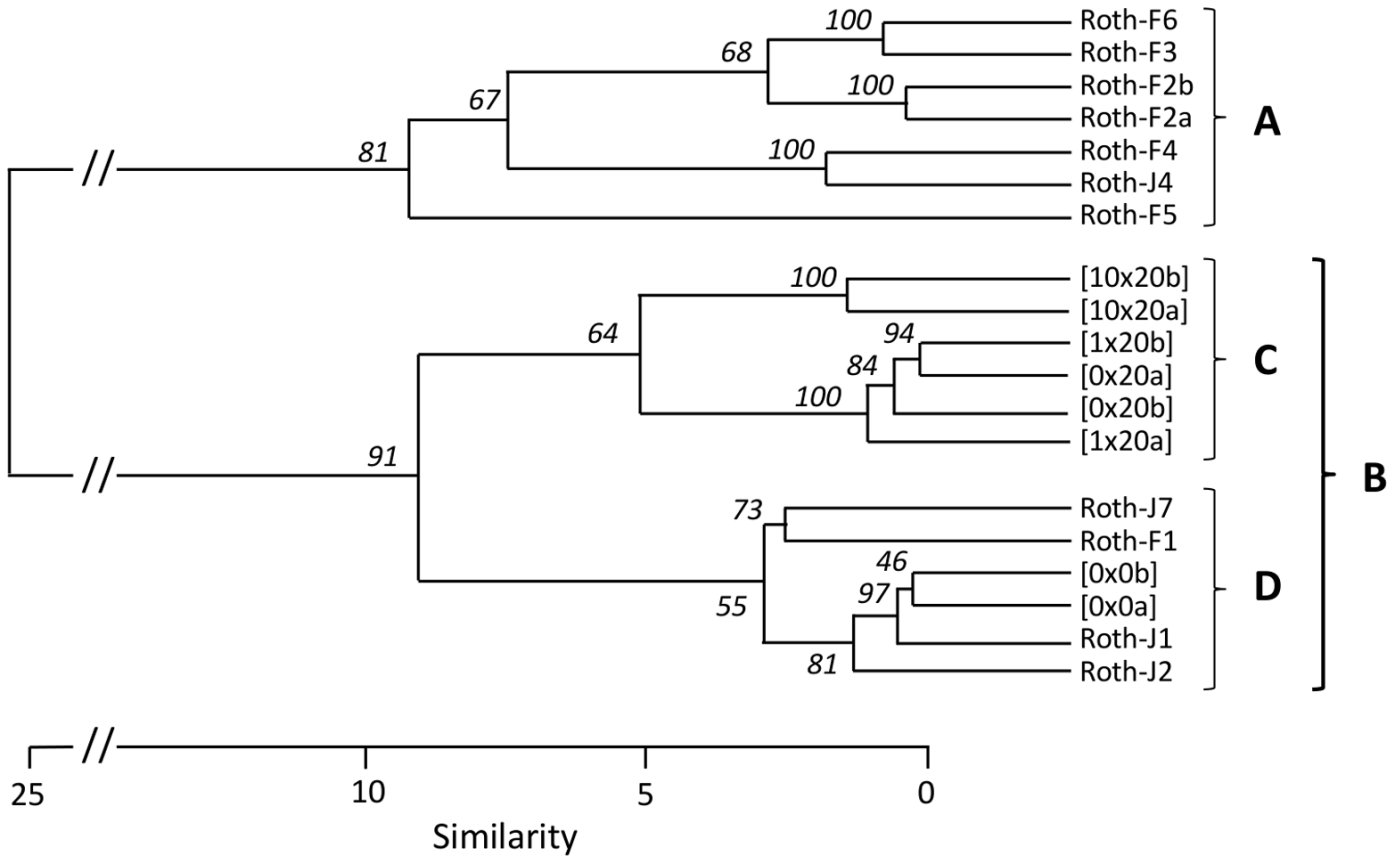
Hierarchical classification of metagenomes used in this study. The dendrogram was established based on taxonomical annotation of reads on M5NR through MG-RAST at the genus level. Clusters were exposed to bootstrap simulation (n = 10000) and bootstrap p-value are indicated at each nod. The grouping was done with the Ward method based on variance analysis, and distances are calculated based on a correlation algorithm (the complement 1*−r* of Pearson’s *r* correlation). Dendrogram scale is representing the similarity based on the Euclidean distance. The letters are indicating the 4 main metagenomic clusters (A: indirect extraction cluster; B: direct extraction cluster; C: microcosm incubation cluster; D: untreated control soil).

**Figure 4:**
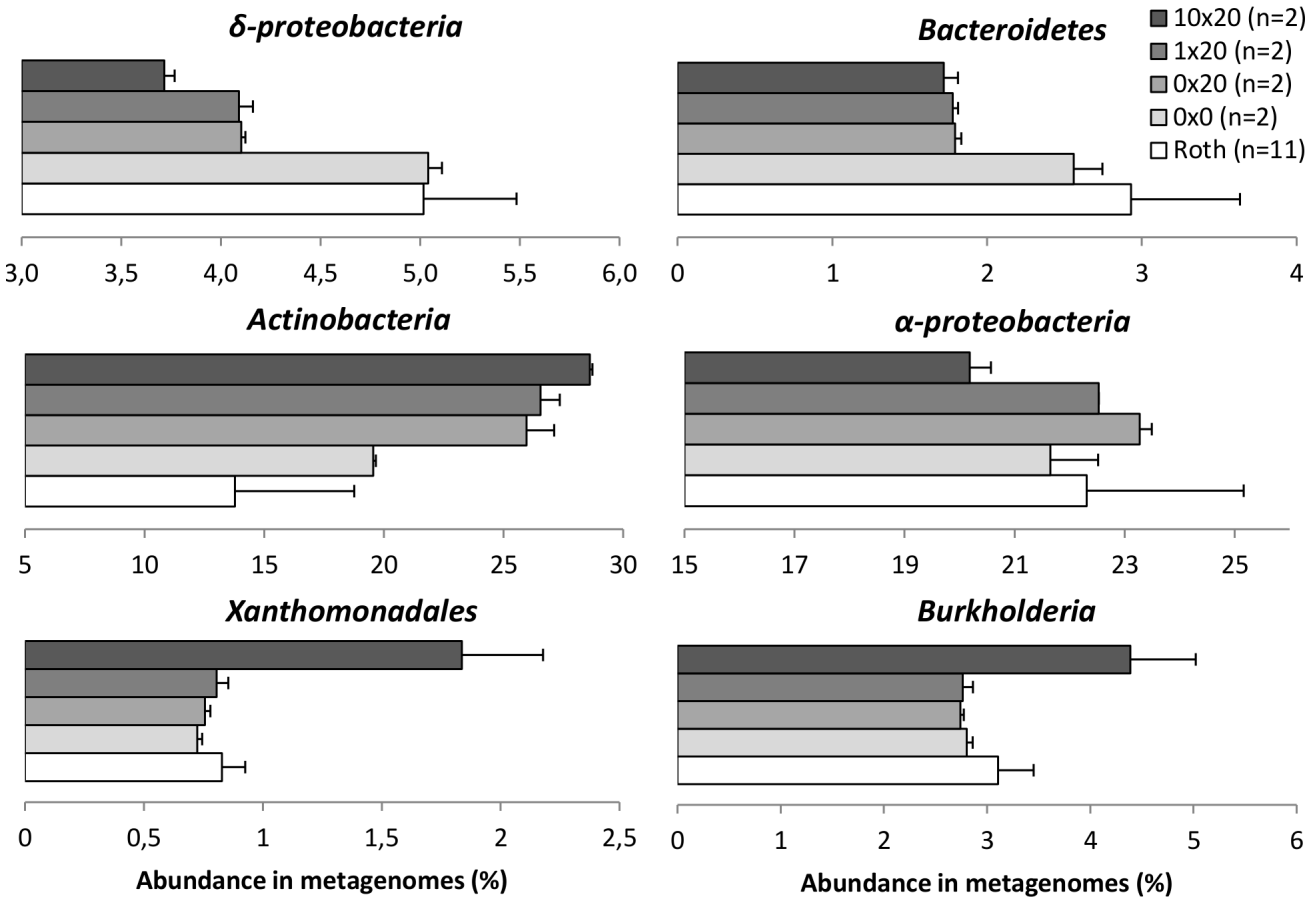
Major taxonomical changes in the soil bacterial community after incubation and chitin enrichment. The graphics represent the abundance of different bacterial groups in percentage of sequences in metagenomes. Grey nuances represent the different conditions and the number of metagenome used is given in brackets. In the case of 0×20, 1×20 and 10×20, the error bars are representing the minimum and maximum observed for each of the two replicates.

**Figure 5:**
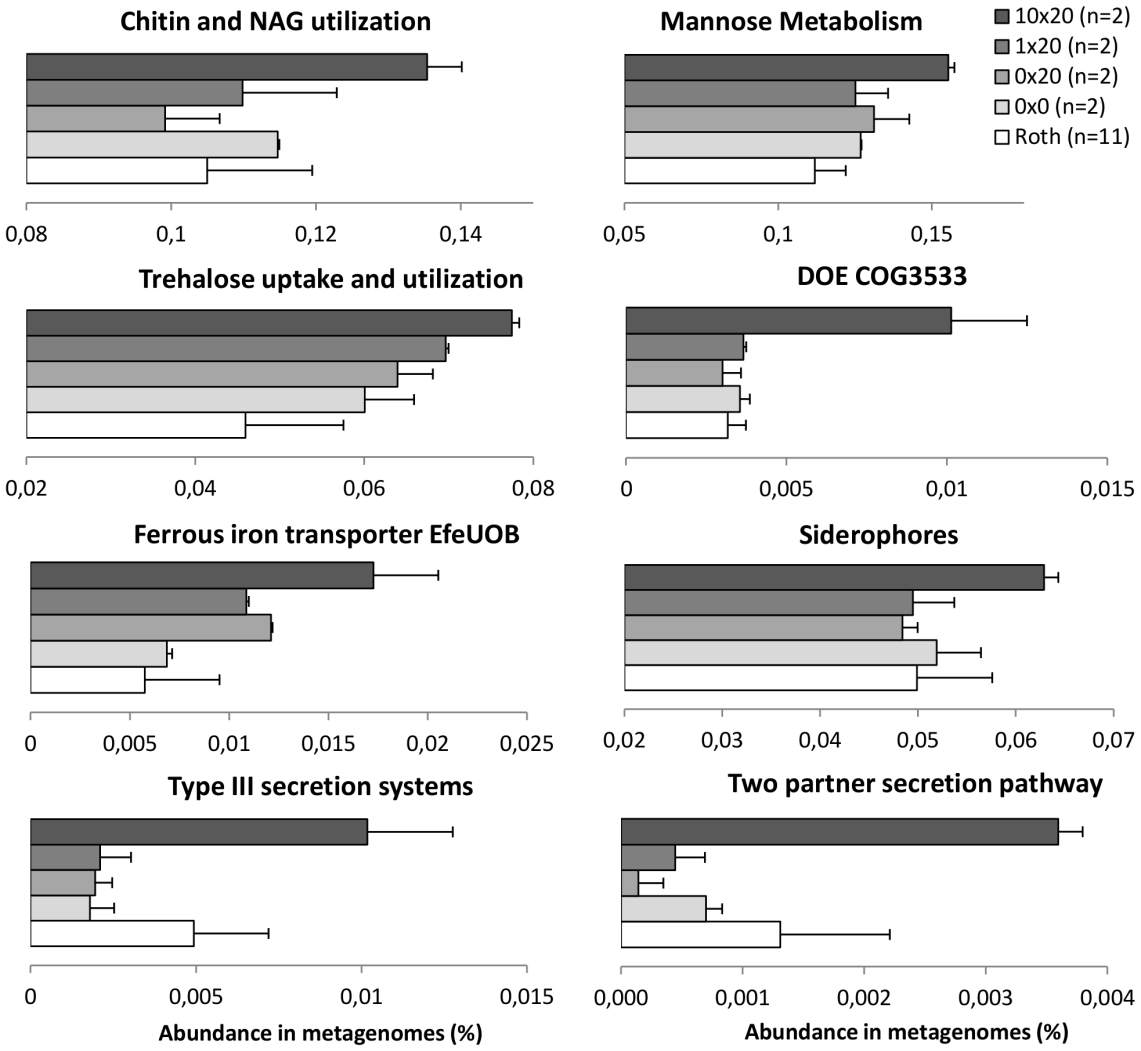
Major functional changes in the soil bacterial community after incubation and chitin enrichment. The graphics represent the abundance of different functional subsystems in percentage of sequences in metagenomes (SEED subsystems classification). Grey nuances represent the different conditions and the number of metagenome used is given in brackets. In the case of 0×20, 1×20 and 10×20, the error bars are representing the minimum and maximum observed for each of the two replicates.

**Figure 6:**
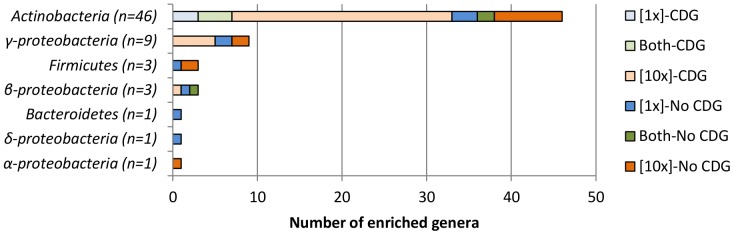
Bacterial genera enriched by chitin treatment. The graphic displays the number of genera belonging to each bacterial group that were increased in abundance in metagenomic datasets after low chitin treatment 1×(blue color nuances), high chitin treatment 10×(orange color nuances), or in both concentration (green nuances). Lighter color nuances indicate the count of genera with known representative species with chitin degrading genes in CAZy (CDG), while darker nuances are representing the ones with no references yet in CAZy (No CDG).

For comparison purposes, 13 pyrosequencing runs obtained from the same soil were integrated to reinforce the analysis [33]. These metagenomes are considered as a reference metagenomic database (annotated as Roth within the text) from the Park Grass soil at Rothamsted research station (Harpenden, UK), including the actual variation imputed to seasons, depth and DNA extraction protocols (Tab. 1). All annotated metagenomes used in this study were submitted to a clusterization-based method in order to hierarchically organize the factors structuring the taxonomical profiles of the soil bacterial community (Fig. 3). The method used for DNA extraction is clearly responsible of the first dichotomy (Fig. 3, clusters A and B), with on one side metagenomes extracted by the “indirect” approach (bacterial cell separation from the soil matrix before lysis, cluster A) and on the other side those obtained by a direct technique (*in situ* lysis of cells, cluster B). This last group includes the 6 metagenomes generated in this study: 0×20a/b, 1×20a/b and 10×20a/b, as well as the two initial untreated control metagenomes 0×0a/b, and also 4 other metagenomes extracted with the same protocol (Tab. 1). The processing of soil in microcosms is responsible for the second dichotomy within cluster B, highlighting again the strong microcosm effect detected earlier (Fig. 3, clusters C: incubated samples; and cluster D: untreated soil samples). Finally, at a third level, chitin enriched metagenomes 10×20 were separated from the two other microcosms conditions 0×20 and 1×20. 10×20a/b duplicated metagenomes clustered adequately, while 1×20b chitin amended soil and incubated control soil 0×20a/b displayed a somewhat similar pattern, indicating that these 3 metagenomes are very similar. Metagenome 1×20a is displaying an intermediary pattern, with higher similarities shared with 0×20a/b and 1×20b cluster. All the conditions used in this study, including Roth, 0×0, 0×20 and 10×20 were statistically compared together through a Welch’s test, except for the 1×20 metagenomes as only few differences were observed between this condition and the incubation control 0×20 (Fig. 3). Three level of analysis were considered considering all the conditions available: (1) analysis at *sensu stricto*, which includes only the incubation control 0×20 and the chitin enriched 10×20; (2) analysis at *sensu medio*, including the non-treated samples 0×0 for investigation of the microcosm effect; and (3) analysis at *sensu lato*, considering all the metagenomes available in the Rothamsted database Roth. Statistical comparison of the metagenomes at the different analytic levels (*sensu stricto*, *sensu medio* and *sensu lato)* is presented in Supplemental Data (File S4) respectively at: the genus level and the lowest functional level from SEED. Major changes at the taxonomical and functional levels are respectively displayed in Fig. 4 and Fig. 5. As a result of this multi-level metagenomic analysis, we proposed a list of all genera reduced in terms of abundance, but known to have chitin degrading representatives (Table S1). At the opposite, we also proposed a list of all genera enriched by chitin treatment in terms of relative abundance in metagenomes (Fig. 6 and Table S2).

## Discussion

### Factors Structuring Metagenomic Studies of Soil Bacterial Communities

The integrative metagenomic approach applied in this study allowed to classify the driving factors impacting the soil bacterial community structure. Based on the pyrosequencing data, the DNA extraction protocol was found to generate the main source of variation between all investigated metagenomes, prior to any other factors, including sampling season and depth, microcosm effect and chitin treatment (Fig. 3, cluster A and B). These observations are coherent with previous published studies, already pointing out the importance of the DNA extraction procedure in microbial ecology studies [51], [52]. This is mostly due to the ability for some species to resist the membrane disruption treatment, which can differ depending on DNA extraction protocols [52]. Furthermore, this effect was already demonstrated for the same soil in previous studies [33], [44], [53].

The second strongest structuring force is attributed to the way soil was processed. All the metagenomes extracted from incubated soil samples (Fig. 3, cluster C) clustered apart from the initial untreated soil metagenomes (Fig. 3, cluster D) highlighting a “microcosm effect”. This is coherent with 16S rRNA qPCR and enzymatic assays which already pointed out the impact of incubation on the soil bacterial community. As temperature is known to be an important factor for chitinase activity [30] [31], it was hypothesized that the temperature difference between incubation (24°C) and the field (15°C) could be involved as part of the detected “microcosm effect”. Interestingly, faster recovery occurred under the highest chitin concentration, probably due to an increase in bacterial growth favored by chitin degradation. This is consistent with the enzymatic assays that show a clear activity peak between day 3 and 20. These results are also coherent with previous published data based on pure culture, reporting a chitinase activity peak after 5 days incubation of *Streptomyces griseus* HUT 6037 with colloidal chitin [34]. The impact of microcosm processing on bacterial community has been already reported [54], and similarly we found a relative increase of *α-proteobacteria*, but we did not confirm the stimulation of *Acidobacteria*. However, our study clearly indicates that one of the main changes in the bacterial community was the increase of *Actinobacteria* under microcosm conditions (Fig. 4). This is probably due to an activation of dormant cells after addition of water during microcosms setting, which might have stimulated spore germination [55].

### Global Impact of Two Chitin Concentrations on Soil Bacterial Communities

Nevertheless, the “microcosm effect” and the DNA extraction biases did not compromise the interest of the approach for exploring the changes occurring in the bacterial community after chitin enrichment. Indeed, the effect of chitin treatment is classified as the third force impacting on the bacterial community, based on the hierarchical classification of metagenomes (Fig. 3, cluster C). At this stage, the lowest chitin concentration was not found to significantly modify the soil bacterial community structure. This is consistent with the other techniques where only small differences were observed in spite of the biostimulant effect observed at day 3 by 16S rRNA qPCR and the diversity increase on phylochips. This concentration was usually applied in previous enrichment based studies [28], [29]. The slight differences observed here could be due to inherent specificities of the soil, or a perturbation of the chitin degradation potential under microcosm conditions, as shown by the enzymatic assays. However, phylochips were found to be more sensitive in detecting the minor community changes occurring under the lowest chitin concentration (Tab. 2). For instance, the low chitin treatment 1× has a clear effect on the observed taxonomical profile at day 10, as well as a noticeable biostimulating effect at day 20 (File S3). This would suggest that the bacterial community shift occurred earlier for the low chitin treated condition. On the other hand, the 10× chitin enrichment resulted also in a clear biostimulation of the bacterial community, as shown by 16S rRNA qPCR, and the concomitant detection of higher genera counts on phylochips (Tab. 2).

### Taxonomic Changes Driven by Chitin in Soil Bacterial Communities

The integrative metagenomic approach resulted in identification of 53 and 12 bacteria genera whose occurrence was noticeably increased by the strongest and the lowest chitin concentration, respectively (Fig. 6 and Table S2). The list includes *γ-proteobacteria* genera, mostly *Xanthomonadales* related genera known to harbor complex glycoside degrading representatives, such as *Xanthomonas* [13], *Xylella* [56], *Lysobacter* [57], *Pseudoxanthomonas* [58] and *Stenotrophomonas* [59]. As observed on phylochips, the strong rise of *Burkholderia* related species, also known for their chitin degrading potential [12], mostly explains the increase of *β-proteobacteria*. In spite of the huge increase of *Actinobacteria* due to microcosm effect, a real selection occurred under high chitin concentration, resulting in an important proportion increase for only 37.7% of the *Actinobacteria* related genera detected in this study (46/122, Fig. 6). This was confirmed by the decrease of the hybridization signals on phylochips corresponding to this phylum, concomitantly to the unique occurence of 4 genera under highest chitin dose: *Aeromicrobium*, *Microbacterium*, *Nocardioides*, *Solirubrobacter* (Tab. 2). Some of this *Actinobacteria*-related genera are already known to have species involved in complex glycoside degradation (e.g. chitin and cellulose), like *Rhodococcus* and *Saccharomonospora* [60], *Actinoplanes*, *Saccharopolyspora* and *Nocardioides* [61], *Amycolatopsis* [62], *Arthrobacter* [63] *Kitasatospora* [11], *Micromonospora* [64], *Mycobacterium* [65], *Nocardia* [66], *Streptosporangium* [67], *Thermomonospora* [68] and *Cellulomonas* [69]. Some of them, like *Aeromicrobium* [70] and *Micrococcus* [57], were reported, even if no species belonging to these genera were yet discovered with chitin degradation genes referenced in CAZy (Table S2). Opposite selection phenomenon was also observed with other taxa containing representatives known to possess a genetic potential for complex biopolymer degradation. For instance, *δ-proteobacteria-related* genera *Geobacter* [71], *Pelobacter* [72], *Desulfovibrio* [73], *ε-proteobacteria*-related genera *Helicobacter* [74], *Arcobacter* [75] and *Bacteroidetes*-related genus *Rhodothermus* [76] displayed lower occurrence under 10× chitin enriched conditions (Table S1).

Even though phylochips are known to be extremely biased toward the non-homogeneous 16S rRNA gene copy numbers in bacterial genomes [37], the double approach used is this study coupling phylochips and high throughput pyrosequencing yielded coherent results. A stronger impact was detected with the 10× concentration on the soil bacterial community at the taxonomic and functional levels, showing higher taxa specialization.

### New Potential Chitin Degraders in the Enriched Soil Microcosms

The main objective of such an enrichment approach combined with a multi-step metagenome analysis was to determine if chitin degraders other than those already isolated could be enhanced in the soil matrix, either for subsequent isolation attempts or direct cloning of their catabolic genes. The study identified several candidate genera with an increased occurrence in chitin treated soil, and yet not reported in the literature for being related to chitin degradation (Table S2). For instance, several genera related to the *Micrococcineae* (sub-order of *Actinobacteria*) were detected under high chitin conditions, including *Renibacterium, Micrococcus, Janibacter, Sanguibacter, Jonesia, Beutenbergia, Kocuria, Cellulomonas, Dermacoccus, Intrasporangium, Xylanimonas, Rothia* and *Arthrobacter*. The list also includes *Cohnella* (*Firmicutes*), *Ectothiorhodospira* and *Thiocystis* (*γ-proteobacteria*), while genera *Lechevalieria* and *Pimelobacter* (*Actinobacteria*), *Terrimonas* (*Bacteroidetes*) and *Tepidimonas* (*β-proteobacteria*) belong to the group of microorganisms responding only at the lowest chitin concentration (Fig. 6 and Table S2). The enrichment approach reveals the presence of individuals related to genera that could be classified as members of the soil rare biosphere based on their relatively low occurrence in natural soil metagenomes. These genera include *Seinonella* (*Firmicutes*), *Williamsia*, *Thermoleophilum* and *Rhodocista* (*Actinobacteria*) in the 10×20 metagenomes, as well as *Okibacterium* (*Actinobacteria*), *Geopsychrobacter* (*δ-proteobacteria*), *Cohnella* [77] and *Thermacetogenium* (*Firmicutes*), *Volucribacter* and *Thioalkalimicrobium* (*γ-proteobacteria*) in 1×20 metagenomes (Table S2). Their detection under enriched conditions indicates that their growth was noticeably enhanced by chitin, strengthening the hypothesis of an increased fitness related to passive or active involvement in chitin degradation. As a consequence, they also contribute to the list of potential candidates for new enzymes since none of these genera is known to contain chitin degraders. However, as chitin degradation is an extra-cellular phenomenon, involvement of bacterial cheaters feeding on chitin degradation products should also be considered [78]. In fact, as the cheaters would tend to be co-selected directly at the beginning of chitin degradation, it is indeed difficult to dissociate the actual degraders from the others, even during early stages of the enrichment kinetic. Nevertheless, it can be hypothesized that bacterial candidates identified in this study are part of a complex bacterial consortium involved in chitin degradation, with probable establishment of biofilm structures upon the chitin fibers. This observation is coherent with literature, as it is known that full degradation of environmental chitin fibers involves a consortium of interacting species [79].

### Impact of Enrichment at the Functional Level

At the functional level, differences observed are more subtle than the ones detected at the taxonomical level. In fact, the global functional profiles of metagenomes are very similar across all conditions when observed at the highest classification level. This can easily be explained by the fact that incubation and enrichment are contributing to select adapted genomes and their associated genes and functions, which are for the major part redundant when classified in an arbitrary database such as SEED. However, at a lower functional subsystem level, specific features were selected by enrichment (Fig. 5). For example, the “Chitin and N-acetylglucosamine (NAG)” subsystem was increased in chitin-amended metagenomes when compared to the controls, as well as many subsystems related to carbon metabolism (e.g. Mannose and trehalose metabolisms). This subsystem includes all the genes involved in chitin and NAG degradation and utilization, indicating that the entire metagenomic pathway was increased after enrichment. This increase was relative and represents only a tiny fraction of the sequenced metagenomes (from 0.1% up to 0.14%, Fig. 5). Even if enrichment resulted in a noticeable, but limited, increase of sequences related to chitin degradation in metagenomes, it might not reflect the true community shift due to shotgun sequencing detection limits. Nevertheless, this augmentation in chitin enriched metagenomes was still enough to detect sequences affiliated to genera that were not detected elsewhere in the analysis within this particular subsystem. For example, sequences of genes involved in chitin degradation could be affiliated to 16 genera only detected in chitin-enriched metagenomes (respectively 8 at 1×: *Acetobacter, Isoptericola, Comamonas, Waddlia, Raphidiopsis, Weissella, Chromohalobacter* and an unclassified *Bacteroidetes* genus; and 8 at 10×: *Hyphomicrobium, Dermacoccus, Bacteriovorax, Sulfurimonas, Ethanoligenens, Streptobacillus, Leptospirillum and an* unclassified *Firmicutes*), while only 5 were retrieved from the incubation control 0×20 (*Raphidiopsis, Halorhodospira, Ewingella, Dietzia, Aeromicrobium*). Unfortunately, only partial sequences were retrieved due to insufficient read length and coverage, limiting the conclusions toward potential new gene discovery. Still, these reads suggest the presence of bacterial genomes closely related to these genera with potential metabolic pathways for chitin and NAG utilization. Furthermore, most of these genera are not yet referenced in the CAZy database for having species with genes involved in chitin degradation. This reinforced the idea that chitin treatment has selected a consortium of unknown species harboring genes related to chitin degradation and utilization.

Some of the secretion systems were increased in chitin 10×20 treatment, including type III secretion systems (T3S) and two partner secretion pathway (TPS). T3S are present in most pathogenic Gram-negative bacteria and are involved in motility and pathogenicity [80], while TPS are involved in many interactions between bacteria and their habitat (e.g. aggregation and biofilm formation, iron acquisition, pathogenicity…) [81]. Concomitantly, an unknown secreted and conserved protein referenced as DOE COG3533 was increased. This protein is apparently affiliated to plant-bacteria interactions with similarities to glycoside hydrolase enzymes based on the SEED classification. In addition, strong chitin concentration resulted in an enrichment of genes involved in iron metabolism, as revealed by the increase of the low pH induced iron transporter EfeUOB and siderophore synthesis subsystems. Iron metabolism is known to be stimulated under aerobic condition as a limiting factor for primary metabolism [82]. Complementary experiments need to be carried out to determine whether if these functions are co-expressed and involved in the selection of their host directly, or an artifact generated by co-selection of genetic resources carried on selected genomes.

## Conclusion

Enrichment in microcosm is a well-known strategy to increase the proportion of micro-organisms reacting to regulated conditions [83], [84], [85]. The selection of chitin-degrading microorganisms confirmed the interest of enrichment for enhancing the proportion of their genes in metagenomes. However, this increase remains relatively low, thus still requiring consequent clone libraries in order to actually notice the enrichment at the sequence level. Our results suggest that the bacterial diversity was increased after chitin treatment, both at low and high concentrations, with a clear selection among known chitin degraders, certainly in favor of the most efficient ones. In addition, novel genera that were not yet reported to be involved in chitin degradation were also selected, indicating that they might be involved in the process of chitin degradation as active members (e.g. degraders, helpers…), or as passive members (e.g. cheaters).

## Supporting Information

Table S1 **Negative genera table.**
(DOCX)

Table S2 **Positive genera table.**
(DOCX)

File S1 **Flowchart of the study.**
(DOCX)

File S2 **RISA and description.**
(DOCX)

File S3 **Phylochips and description.**
(DOCX)

File S4 **Metagenomic analysis and description.**
(DOCX)

## References

[1] Gomes RC, Semedo LT, Soares RM, Linhares LF, Ulhoa CJ, et al (2001) Purification of a thermostable endochitinases from *Streptomyces RC1071* isolated from a cerrado soil and its antagonism against phytopathogenic fungi. J Appl Microbiol 90: 653–661. 11309080 10.1046/j.1365-2672.2001.01294.x

[2] LeCleir GR, Buchan A, Maurer J, Moran MA, Hollibaugh JT (2007) Comparison of chitinolytic enzymes from an alkaline hypersaline lake and an estuary. Environ Microb 9: 197–205. 10.1111/j.1462-2920.2006.01128.x 17227424

[3] Muzzarelli RA (1999) Native, Industrial, and fossil chitin. EXS 87: 1–6. 10906948 10.1007/978-3-0348-8757-1_1

[4] Williamson N, Brian P, Wellington EM (2000) Molecular detection of bacterial and *streptomycete* chitinases in the environment. Antonie van Leeuwenhoek 78: 315–321. 11386354 10.1023/a:1010225909148

[5] Metcalfe AC, Krsek M, Gooday GW, Prosser JI, Wellington EM (2002) Molecular analysis of bacterial chitinolytic community in an upland pasture. Appl Environ Microbiol 68: 5042–5050. 12324355 10.1128/AEM.68.10.5042-5050.2002 PMC126395

[6] LeCleir GR, Buchan A, Hollibaugh JT (2004) Chitinase gene sequences retrieved from diverse aquatic habitats reveal environment-specific distributions. Appl Environ Microbiol 70: 6977–6983. 15574890 10.1128/AEM.70.12.6977-6983.2004 PMC535185

[7] Ramaiah N, Hill RT, Chun J, Ravel J, Matte MH, et al (2000) Use of chiA probe for detection of chitinases genes in bacteria from Chesapeake Bay. FEMS Microbiol Ecol 34: 63–71. 11053737 10.1111/j.1574-6941.2000.tb00755.x

[8] Hobel CF, Marteinsson VT, Hreggvidsson GO, Kristjansson JK (2005) Investigation of the microbial ecology of intertidal hot springs by using diversity analysis of 16S rRNA and chitinases genes. Appl Environ Microbiol 71: 2771–2776. 15870372 10.1128/AEM.71.5.2771-2776.2005 PMC1087530

[9] Xiao X, Yin X, Lin J, Sun L, You Z, et al (2005) Chitinase Genes in Lake Sediments of Ardley Island, Antartica. Appl Environ Microbiol 71: 7904–7909. 16332766 10.1128/AEM.71.12.7904-7909.2005 PMC1317360

[10] Howard M, Ekborg N, Taylor II L, Weiner R, Hutcheson S (2003) Chitinase B of “*Microbulbifer degradans*” 2–40 contains two catalytic domains with different chitinolytic activities. J Bacteriol 186: 1297–1303. 10.1128/JB.186.5.1297-1303.2004 PMC344425 14973034

[11] Hjort K, Bergström M, Adesina M, Jansson J, Smalla K, et al (2009) Chitinases genes revealed and compared in bacterial isolates, DNA extracts and a metagenomic library from a phytopathogen-suppressive soil. FEMS Microbiol Ecol 71: 197–207. 19922433 10.1111/j.1574-6941.2009.00801.x

[12] Ogawa K, Yoshida N, Kariya K, Ohnishi C, Ikeda R (2002) Purification and characterization of a novel chitinase from *Burkholderia cepacia strain KH2* isolated from the bed log of Lentinus edodes, Shiitake mushroom. J Gen Appl Microbiol 48: 25–33. 12469313 10.2323/jgam.48.25

[13] Yamaoka H, Hayashi H, Karita S, Kimura T, Sakka K, et al (1999) Purification and some properties of a chitinase from *Xanthomonas sp*. *strain AK* . J Biosci Bioeng 88: 328–330. 16232621 10.1016/s1389-1723(00)80019-5

[14] Gooday GW (1990) The ecology of chitin degradation. Adv Microb Ecol 11: 387–430.

[15] Beier S, Bertilsson S (2013) Bacterial chitin degradation-mechanisms and ecophysiological strategies. Front Microbiol 4: 149. 23785358 10.3389/fmicb.2013.00149 PMC3682446

[16] Horn SJ, Sikorski P, Cederkvist JB, Vaaje-Kolstad G, Sørlie M, et al (2006) Costs and benefits of processivity in enzymatic degradation of recalcitrant polysaccharides. Proc Natl Acad Sci USA 103: 18089–94. 17116887 10.1073/pnas.0608909103 PMC1838711

[17] Han SJ, Park H, Lee SG, Lee HK, Yim JH (2011) Optimization of cold-active chitinase production from the Antarctic bacterium, *KOPRI 21702* . Appl Microbiol Biotechnol 89: 613–621. 20922383 10.1007/s00253-010-2890-y

[18] Cantarel BL, Coutinho PM, Rancurel C, Bernard T, Lombard V et al.. (2009) The Carbohydrate-Active EnZymes database (CAZy): an expert resource for Glycogenomics. Nucleic Acids Res 37(Database issue): D233–238. 10.1093/nar/gkn663 PMC2686590 18838391

[19] Henrissat B (1991) A classification of glycosyl hydrolases based on amino acid sequence similarities. Biochem J 280: 309–316. 1747104 10.1042/bj2800309 PMC1130547

[20] Henrissat B, Bairoch A (1993) New families in the classification of glycosyl hydrolases based on amino acid sequence similarities. Biochem J 293: 781–788. 8352747 10.1042/bj2930781 PMC1134435

[21] Vaaje-Kolstad G, Westereng B, Horn SJ, Liu Z, Zhai H, et al (2010) An oxidative enzyme boosting the enzymatic conversion of recalcitrant polysaccharides. Science 330: 219–222. 20929773 10.1126/science.1192231

[22] Lombard V, Bernard T, Rancurel C, Brumer H, Coutinho PM, et al (2010) A hierarchical classification of polysaccharide lyases for glycogenomics. Biochem J 432: 437–444. 20925655 10.1042/BJ20101185

[23] Graham LS, Sticklen MB (1994) Plant chitinases. Can J Bota 72: 1057–1083.

[24] Harman GE, Hayes CK, Lorito M, Broadway RM, diPietro A, et al (1993) Chitinolytic enzymes of *Trichoderma harzianum*: purification of chitobiosidase and endochitinase. Phytopathol 83: 313–318.

[25] Cottrell MT, Moore JA, Kirchman DL (1999) Chitinases from uncultured marine microorganisms. Appl Environ Microbiol 65: 2553–2557. 10347042 10.1128/aem.65.6.2553-2557.1999 PMC91377

[26] Kielak AM, Cretoiu MS, Semenov AV, Sørensen SJ, van Elsas JD (2013) Bacterial chitinolytic communities respond to chitin and pH alteration in soil. Appl Environ Microbiol 79: 263–272. 23104407 10.1128/AEM.02546-12 PMC3536121

[27] Bhuiyan FA, Nagata S, Ohnishi K (2011) Novel chitinase genes from metagenomic DNA prepared from marine sediments in southwest Japan. Pak J Biol Sci 14: 204–211. 21870643 10.3923/pjbs.2011.204.211

[28] Inglis GD, Kawchuk LM (2002) Comparative degradation of *oomycete*, *ascomycete*, and *basidiomycete* cell walls by mycoparasitic and biocontrol fungi. Can J Microbiol 48: 60–70. 11888164 10.1139/w01-130

[29] Hjort K, Lembke A, Speksnijder A, Smalla K, Jansson JK (2007) Community structure of actively growing bacterial populations in plant pathogen suppressive soil. Microb Ecol 53: 399–413. 16944345 10.1007/s00248-006-9120-2

[30] Vorob’ev AV, Manucharova NA, Iaroslavtsev AM, Belova EV, Zviagintsev DG, et al (2007) The composition of the chitinolytic microbial complex and its effect on chitin decomposition at various humidity levels. Mikrobiologiia 76: 632–638. 18069323

[31] Manucharova NA, Vlasenko AN, Men’ko EV, Zviagintsev DG (2011) Specificity of the chitinolytic microbial complex of soils incubated at different temperatures. Mikrobiologiia 80: 219–229. 21774192

[32] Vogel TM, Simonet P, Jansson JK, Hirsch PR, Tiedje JM, et al (2009) TerraGenome: a consortium for the sequencing of a soil metagenome. Nat. Rev. Microbiol 7: 252.

[33] Delmont TO, Prestat E, Keegan KP, Faubladier M, Robe P, et al (2012) Structure, fluctuation and magnitude of a natural grassland soil metagenome. ISME J 6: 1677–1687. 22297556 10.1038/ismej.2011.197 PMC3498926

[34] FAO (2006) Guidelines for soil description. FAO, Rome, Italy. ftp://ftp.fao.org/agl/agll/docs/guidel_soil_descr.pdf. Accessed 2013 Jun 3.

[35] Kwang K, Hong-Seok J (2001) Effect of Chitin Sources on Production of Chitinase and Chitosanase by *Streptomyces griseus HUT 6037*. Biotechnol. Bioprocess Eng 6: 18–24.

[36] Ren Y, Wee KE, Chang FN (2000) Deficiency of current methods in assaying endochitinase activity. Biochem Biophys Res Commun 268: 302–305. 10679198 10.1006/bbrc.2000.2118

[37] Griffiths RI, Whitely AS, O’Donnell AG, Bailey MJ (2000) Rapid method for coextraction of DNA and rRNA from natural environments for analysis of ribosomal DNA- and rRNA-based microbial community composition. Appl Environ Microbiol 66: 5488–5491. 11097934 10.1128/aem.66.12.5488-5491.2000 PMC92488

[38] Ranjard L, Poly F, Lata JC, Mougel C, Thioulouse J, et al (2001) Characterization of bacterial and fungal soil communities by automated ribosomal intergenic spacer analysis fingerprints: biological and methodological variability. Appl Environ Microbiol 67: 4479–4487. 11571146 10.1128/AEM.67.10.4479-4487.2001 PMC93193

[39] Fierer N, Jackson JA, Vilgalys R, Jackson RB (2005) Assessment of soil microbial community structure by use of taxon-specific quantitative PCR assays. Appl Environ Microbiol 71: 4117–4120. 16000830 10.1128/AEM.71.7.4117-4120.2005 PMC1169028

[40] Klappenbach JA, Dunbar JM, Schmidt TM (2000) rRNA operon copy number reflects ecological strategies of bacteria. Appl Environ Microbiol 66: 1328–1333. 10742207 10.1128/aem.66.4.1328-1333.2000 PMC91988

[41] Martin-Laurent F, Philippot L, Hallet S, Chaussod R, Germon JC, et al (2001) DNA extraction from soils: old bias for new microbial diversity analysis methods. Appl Environ Microbiol 67: 2354–2359. 11319122 10.1128/AEM.67.5.2354-2359.2001 PMC92877

[42] Bruce KD, Hiorns WD, Hobman JL, Osborn AM, Strike P, et al (1992) Amplification of DNA from native populations of soil bacteria by using the polymerase chain reaction. Appl Environ Microbiol 58: 3413–3416. 1444376 10.1128/aem.58.10.3413-3416.1992 PMC183114

[43] Sanguin H, Remenant B, Dechesne A, Thioulouse J, Vogel TM, et al (2006) Potential of a 16S rRNA-based taxonomic microarray for analyzing the rhizosphere effects of maize on *Agrobacterium spp*. and bacterial communities. Appl Environ Microbiol 72: 4302–4312. 16751545 10.1128/AEM.02686-05 PMC1489601

[44] Delmont TO, Robe P, Cecillon S, Clark IM, Constancias F, et al (2011) Accessing the soil metagenome for studies of microbial diversity. Appl Environ Microbiol 77: 1315–1324. 21183646 10.1128/AEM.01526-10 PMC3067229

[45] Niu B, Fu L, Sun S, Li W (2010) Artificial and natural duplicates in pyrosequencing reads of metagenomic data. BMC Bioinformatics 11: 187. 20388221 10.1186/1471-2105-11-187 PMC2874554

[46] Meyer F, Paarmann D, D’Souza M, Olson R, Glass EM (2008) The metagenomics RAST server-a public resource for the automatic phylogenetic and functional analysis of metagenomes. BMC Bioinformatics 9: 386. 18803844 10.1186/1471-2105-9-386 PMC2563014

[47] Aziz RK, Bartels D, Best AA, DeJongh M, Disz T, et al (2008) The RAST Server: rapid annotations using subsystems technology. BMC Genomics 9: 75. 18261238 10.1186/1471-2164-9-75 PMC2265698

[48] R Development Core Team (2011) R: A language and environment for statistical computing. R Foundation for Statistical Computing, Vienna, Austria. ISBN 3-900051-07-0, http://www.R-project.org/. Accessed 2013 Jun 3.

[49] Parks DH, Beiko RG (2010) Identifying biologically relevant differences between metagenomic communities. Bioinformatics 26: 715–721. 20130030 10.1093/bioinformatics/btq041

[50] Klindworth A, Pruesse E, Schweer T, Peplies J, Quast C, et al (2013) Evaluation of general 16S ribosomal RNA gene PCR primers for classical and next-generation sequencing-based diversity studies. Nucleic Acids Res 41: e1. 22933715 10.1093/nar/gks808 PMC3592464

[51] Inceoglu O, Salles JF, Van Overbeek L, Van Elsas JD (2010) Effects of plant genotype and growth stage on the *betaproteobacterial* communities associated with different potato cultivars in two fields. Appl Environ Microbiol 76: 3675–3684. 20363788 10.1128/AEM.00040-10 PMC2876460

[52] Frostegård A, Courtois S, Ramisse V, Clerc S, Bernillon D, et al (1999) Quantification of bias related to the extraction of DNA directly from soils. Appl Environ Microbiol 65: 5409–5420. 10583997 10.1128/aem.65.12.5409-5420.1999 PMC91737

[53] Delmont TO, Robe P, Clark I, Simonet P, Vogel TM (2011) Metagenomic comparison of direct and indirect soil DNA extraction approaches. J Microbiol Methods 86: 397–400. 21723887 10.1016/j.mimet.2011.06.013

[54] Thomson BC, Ostle NJ, McNamara NP, Whiteley AS, Griffiths RI (2010) Effects of sieving, drying and rewetting upon soil bacterial community structure and respiration rates. J Microbiol Methods 83: 69–73. 20691223 10.1016/j.mimet.2010.07.021

[55] Jenkins SN, Waite IS, Blackburn A, Husband R, Rushton SP, et al (2009) Actinobacterial community dynamics in long term managed grasslands. Antonie Van Leeuwenhoek 95: 319–334. 19247797 10.1007/s10482-009-9317-8

[56] Killiny N, Prado SS, Almeida RP (2010) Chitin utilization by the insect-transmitted bacterium *Xylella fastidiosa* . Appl Environ Microbiol 76: 6134–6140. 20656858 10.1128/AEM.01036-10 PMC2937478

[57] Cervantes-González E, Rojas-Avelizapa NG, Cruz-Camarillo R, García-Mena J, Rojas-Avelizapa LI (2008) Oil-removal enhancement in media with keratinous or chitinous wastes by hydrocarbon-degrading bacteria isolated from oil-polluted soils. Environ Technol 29: 171–182. 18613616 10.1080/09593330802028659

[58] Someya N, Ikeda S, Morohoshi T, Noguchi Tsujimoto M, Yoshida T, et al (2011) Diversity of culturable chitinolytic bacteria from rhizospheres of agronomic plants in Japan. Microbes Environ 26: 7–14. 21487197 10.1264/jsme2.me10149

[59] Okeke BC, Lu J (2011) Characterization of a defined cellulolytic and xylanolytic bacterial consortium for bioprocessing of cellulose and hemicelluloses. Appl Biochem Biotechnol 163: 869–881. 20859703 10.1007/s12010-010-9091-0

[60] Steger K, Jarvis A, Vasara T, Romantschuk M, Sundh I (2007) Effects of differing temperature management on development of *Actinobacteria* populations during composting. Res Microbiol 158: 617–624. 17683913 10.1016/j.resmic.2007.05.006

[61] Nawani NN, Kapadnis BP (2003) Chitin degrading potential of bacteria from extreme and moderate environment. Indian J Exp Biol 41: 248–254. 15267156

[62] Saito A, Ooya T, Miyatsuchi D, Fuchigami H, Terakado K, et al (2009) Molecular characterization and antifungal activity of a family 46 chitosanase from *Amycolatopsis sp*. CsO-2. FEMS Microbiol Lett 293: 79–84. 19236484 10.1111/j.1574-6968.2009.01507.x

[63] Mavromatis K, Feller G, Kokkinidis M, Bouriotis V (2003) Cold adaptation of a psychrophilic chitinase: a mutagenesis study. Protein Eng 16: 497–503. 12915727 10.1093/protein/gzg069

[64] Gacto M, Vicente-Soler J, Cansado J, Villa TG (2000) Characterization of an extracellular enzyme system produced by *Micromonospora chalcea* with lytic activity on yeast cells. J Appl Microbiol 88: 961–967. 10849171 10.1046/j.1365-2672.2000.01065.x

[65] Varrot A, Leydier S, Pell G, Macdonald JM, Stick RV, et al (2005) *Mycobacterium tuberculosis* strains possess functional cellulases. J Biol Chem 280: 20181–20184. 15824123 10.1074/jbc.C500142200

[66] Nanjo F, Katsumi R, Sakai K (1990) Purification and characterization of an exo-beta-D-glucosaminidase, a novel type of enzyme, from *Nocardia orientalis* . J Biol Chem 265: 10088–10094. 2351651

[67] Manucharova NA, Belova EV, Polianskaia LM, Zenova GM (2004) A chitinolytic *actinomycete* complex in black soil. Mikrobiologiia 73: 68–72. 15074043

[68] Anish R, Rahman MS, Rao M (2007) Application of cellulases from an alkalothermophilic *Thermomonospora sp*. in biopolishing of denims. Biotechnol Bioeng 96: 48–56. 16952150 10.1002/bit.21175

[69] Reguera G, Leschine SB (2003) Biochemical and genetic characterization of ChiA, the major enzyme component for the solubilization of chitin by *Cellulomonas uda.* . Arch Microbiol 180: 434–443. 14586544 10.1007/s00203-003-0611-y

[70] Lu WJ, Wang HT, Yang SJ, Wang ZC, Nie YF (2005) Isolation and characterization of mesophilic cellulose-degrading bacteria from flower stalks-vegetable waste co-composting system. J Gen Appl Microbiol 51: 353–360. 16474195 10.2323/jgam.51.353

[71] Ren Z, Steinberg LM, Regan JM (2008) Electricity production and microbial biofilm characterization in cellulose-fed microbial fuel cells. Water Sci Technol 58: 617–622. 18725730 10.2166/wst.2008.431

[72] Kiely PD, Rader G, Regan JM, Logan BE (2011) Long-term cathode performance and the microbial communities that develop in microbial fuel cells fed different fermentation endproducts. Bioresour Technol 102: 361–366. 20570144 10.1016/j.biortech.2010.05.017

[73] Kan J, Wang Y, Obraztsova A, Rosen G, Leather J, et al (2011) Marine microbial community response to inorganic and organic sediment amendments in laboratory mesocosms. Ecotoxicol Environ Saf 74: 1931–1941. 21784523 10.1016/j.ecoenv.2011.06.011

[74] Takaishi S, Wang TC (2007) Gene expression profiling in a mouse model of Helicobacter-induced gastric cancer. Cancer Sci 98: 284–293. 17270017 10.1111/j.1349-7006.2007.00392.x PMC11159662

[75] Chen WC, Tseng WN, Hsieh JL, Wang YS, Wang SL (2010) Biodegradation and microbial community changes upon shrimp shell wastes amended in mangrove river sediment. J Environ Sci Health B 45: 473–477. 20512738 10.1080/03601231003800305

[76] Hobel CF, Hreggvidsson GO, Marteinsson VT, Bahrani-Mougeot F, Einarsson JM, et al (2004) Cloning, expression, and characterization of a highly thermostable family 18 chitinase from Rhodothermus marinus. Extremophiles 9: 53–64. 15583965 10.1007/s00792-004-0422-3

[77] Eida MF, Nagaoka T, Wasaki J, Kouno K (2012) Isolation and characterization of cellulose-decomposing bacteria inhabiting sawdust and coffee residue composts. Microbes Environ 27: 226–233. 22353767 10.1264/jsme2.ME11299 PMC4036048

[78] Allison SD (2005) Cheaters, diffusion and nutrients constrain decomposition by microbial enzymes in spatially structured environments. Ecol Lett 8: 626–635.

[79] Jagmann N, von Rekowski KS, Philipp B (2011) Interactions of bacteria with different mechanisms for chitin degradation result in the formation of a mixed-species biofilm. FEMS Microbiol Lett 326: 69–75. 22092834 10.1111/j.1574-6968.2011.02435.x

[80] Büttner D (2012) Protein Export According to Schedule: Architecture, Assembly, and Regulation of Type III Secretion Systems from Plant- and Animal-Pathogenic Bacteria. Microbiol Mol Biol Rev 76: 262–310. 22688814 10.1128/MMBR.05017-11 PMC3372255

[81] Jacob-Dubuisson F, Guérin J, Baelen S, Clantin B (2013) Two-partner secretion: as simple as it sounds? Res Microbiol 164: 583–595. 23542425 10.1016/j.resmic.2013.03.009

[82] Giessen TW, Franke KB, Knappe TA, Kraas FI, Bosello M, et al (2012) Isolation, Structure Elucidation, and Biosynthesis of an Unusual Hydroxamic Acid Ester-Containing Siderophore from *Actinosynnema mirum* . J Nat Prod 75: 905–914. 22578145 10.1021/np300046k

[83] Krsek M, Wellington EM (2001) Assessment of chitin decomposer diversity within an upland grassland. Antonie Van Leeuwenhoek 79: 261–267. 11816968 10.1023/a:1012043401168

[84] Ramírez-Saad HC, Sessitsch A, de Vos WM, Akkermans AD (2000) Bacterial community changes and enrichment of *Burkholderia*-like bacteria induced by chlorinated benzoates in a peat-forest soil-microcosm. Syst Appl Microbiol 23: 591–598. 11249031 10.1016/S0723-2020(00)80035-1

[85] Wagner-Döbler I, Bennasar A, Vancanneyt M, Strömpl C, Brümmer I, et al (1998) Microcosm enrichment of biphenyl-degrading microbial communities from soils and sediments. Appl Environ Microbiol 64: 3014–3022. 9687466 10.1128/aem.64.8.3014-3022.1998 PMC106808

