# Supplementary material for: Soil Bacterial Community Shifts after Chitin Enrichment: An Integrative Metagenomic Approach
Source: PLoS One. 2013 Nov 20;8(11):e79699. doi: 10.1371/journal.pone.0079699 (PMC3835784; doi:10.1371/journal.pone.0079699)
Supplement: Table S1 — Negative genera table. (DOCX) [file pone.0079699.s001.docx]

**Table S1: List of all the genera decreased through enrichment and known to have chitin degrading representatives**

|  |  | **Chitin degrading enzymes (CAZy)** | | | |  |
| --- | --- | --- | --- | --- | --- | --- |
| **Bacterial group** | **Decreased genera** | **AA10** | **CE4** | **GH18** | **GH19** | **References** |
|  | *Geobacter* | - | + | - | + | [71] |
|  | *Pelobacter* | - | + | - | + | [72] |
|  | *Lawsonia* | - | + | - | - |  |
| *δ-proteobacteria* | *Desulfurivibrio* | - | + | - | - |  |
| (n=8) | *Syntrophus* | - | + | - | - |  |
|  | *Desulfobacterium* | - | + | - | - |  |
|  | *Desulfovibrio* | - | + | + | - | [73] |
|  | *Haliangium* | - | + | + | - |  |
|  | *Helicobacter* | - | + | - | + | [74] |
| *ε-proteobacteria* | *Arcobacter* | - | + | - | + | [75] |
| (n=4) | *Campylobacter* | - | + | - | - |  |
|  | *Wolinella* | - | + | - | - |  |
|  | *Prochlorococcus* | - | - | + | - |  |
| *Cyanobacteria* | *Gloeobacter* | - | + | - | - |  |
| (n=3) | *Thermosynechococcus* | - | + | - | - |  |
| *Bacteroidetes* | *Rhodothermus* | - | - | + | - | [76] |
| (n=2) | *Spirosoma* | - | + | + | - |  |
| *Aquificae* | *Sulfurihydrogenibium* | - | + | - | - |  |
| (n=2) | *Thermocrinis* | - | + | - | - |  |
| *Spirochaetes* | *Leptospira* | - | + | + | - |  |
| (n=2) | *Spirochaeta* | - | + | + | - |  |
| *Acidobacteria* | *Fibrobacter* | - | - | + | - |  |
| *Deferribacteres* | *Calditerrivibrio* | - | + | - | - |  |
| *β-proteobacteria* | *Candidatus accumulibacter* | - | + | - | - |  |
| *Verrucomicrobia* | *Opitutus* | - | - | + | - |  |

Table S1: Summarized table of some of the bacterial genera noticeably reduced by high chitin enrichment 10x after comparison with all metagenomic conditions. Only the genera known to have representatives harboring chitin degradation related genes in CAZy are reported (AA10, CE4, GH18 and GH19). The presence of characterized enzymes related to chitin degradation in CAZy for each genus is given, as well as references that reported involvement in degradation of complex carbohydrate molecules such as chitin and cellulose, as some of the characterized chitinases in CAZy were reported to have a catalytic activity toward cellulose.
