# Supplementary material for: Soil Bacterial Community Shifts after Chitin Enrichment: An Integrative Metagenomic Approach
Source: PLoS One. 2013 Nov 20;8(11):e79699. doi: 10.1371/journal.pone.0079699 (PMC3835784; doi:10.1371/journal.pone.0079699)
Supplement: Table S2 — Positive genera table. (DOCX) [file pone.0079699.s002.docx]

**Table S2: Summarized table of bacterial genera selected by chitin enrichment.**

|  | **Chitin** | **Enriched** | **Chitin degrading enzymes (CAZy)** | | | |  |
| --- | --- | --- | --- | --- | --- | --- | --- |
| **Bacterial group** | **treatment** | **genera** | **AA10** | **CE4** | **GH18** | **GH19** | **References** |
|  |  | *Acidimicrobium* | - | - | + | - |  |
|  |  | *Conexibacter* | - | + | + | - |  |
|  | Chitin 1x20 | *Lechevalieria* | - | - | - | - |  |
|  |  | *Okibacterium* | - | - | - | - |  |
|  |  | *Pimelobacter* | - | - | - | - |  |
|  |  | *Saccharomonospora* | - | + | - | - | [55] |
|  |  | *Amycolatopsis* | + | + | + | + | [58] |
|  |  | *Catenulispora* | + | + | + | + |  |
|  | 1x20 and 10x20 | *Cystobacter* | - | - | - | - |  |
|  |  | *Dermacoccus* | - | - | - | - |  |
|  |  | *Kitasatospora* | - | - | - | + | [59] |
|  |  | *Stackebrandtia* | + | - | + | + |  |
|  |  | *Actinoplanes* | + | + | + | + | [56] |
|  |  | *Actinosynnema* | + | + | + | + |  |
|  |  | *Aeromicrobium* | - | - | - | - | [57] |
|  |  | *Arthrobacter* | - | + | + | - | [59] |
|  |  | *Beutenbergia* | - | + | - | - |  |
|  |  | *Cellulomonas* | + | + | + | + | [60] |
|  |  | *Dactylosporangium* | - | - | - | - |  |
|  |  | *Gordonia* | - | + | - | - |  |
|  |  | *Intrasporangium* | - | + | - | - |  |
|  |  | *Janibacter* | - | - | - | - |  |
| ***Actinobacteria*** |  | *Jonesia* | + | + | + | - |  |
| ***(n=46)*** |  | *Kineococcus* | - | - | - | - |  |
|  |  | *Kocuria* | - | - | - | - |  |
|  |  | *Kribbella* | + | + | + | + |  |
|  |  | *Micrococcus* | - | - | - | - | [61] |
|  | Chitin 10x20 | *Micromonospora* | + | + | + | + | [62] |
|  |  | *Mycobacterium* | - | + | + | + | [63] |
|  |  | *Nakamurella* | - | + | + | - |  |
|  |  | *Nocardia* | - | + | - | - | [64] |
|  |  | *Nocardioides* | - | + | + | - | [56] |
|  |  | *Propionibacterium* | - | + | + | - |  |
|  |  | *Renibacterium* | + | + | + | - |  |
|  |  | *Rhodococcus* | - | + | - | + | [55] |
|  |  | *Rothia* | - | + | - | - |  |
|  |  | *Saccharopolyspora* | + | + | + | - | [56] |
|  |  | *Salinispora* | + | + | + | + |  |
|  |  | *Sanguibacter* | + | - | + | - | [24] |
|  |  | *Streptomyces* | + | + | + | + | [29] |
|  |  | *Streptosporangium* | + | + | + | + | [65] |
|  |  | *Thermobispora* | + | + | + | - |  |
|  |  | *Thermoleophilum* | - | - | - | - |  |
|  |  | *Thermomonospora* | + | + | - | - | [66] |
|  |  | *Williamsia* | - | - | - | - |  |
|  |  | *Xylanimonas* | + | + | + | - |  |
|  | Chitin 1x20 | *Thioalkalimicrobium* | - | - | - | - |  |
|  |  | *Volucribacter* | - | - | - | - |  |
|  |  | *Ectothiorhodospira* | - | - | - | - |  |
| ***γ-proteobacteria*** |  | *Lysobacter* | - | - | + | - | [61] |
| ***(n=9)*** |  | *Pseudoxanthomonas* | - | + | - | - | [67] |
|  | Chitin 10x20 | *Stenotrophomonas* | + | + | + | + | [68] |
|  |  | *Thiocystis* | - | - | - | - |  |
|  |  | *Xanthomonas* | - | + | + | + | [13] |
|  |  | *Xylella* | - | + | + | - | [69] |
|  | Chitin 1x20 | *Tepidimonas* | - | - | - | - |  |
| ***β-proteobacteria*** | 1x20 and 10x20 | *Zoogloea* | - | - | - | - |  |
| ***(n=3)*** | Chitin 10x20 | *Burkholderia* | + | + | + | + | [12] |
|  | Chitin 1x20 | *Thermacetogenium* | - | - | - | - |  |
| ***Firmicutes (n=3)*** | Chitin 10x20 | *Cohnella* | - | - | - | - | [77] |
|  |  | *Seinonella* | - | - | - | - |  |
| **α*-proteobacteria*** | Chitin 10x20 | *Rhodocista* | - | - | - | - |  |
| **δ*-proteobacteria*** | Chitin 1x20 | *Geopsychrobacter* | - | - | - | - |  |
| ***Bacteroidetes*** | Chitin 1x20 | *Terrimonas* | - | - | - | - |  |

Table S2: Summarized table of the bacterial genera selected by chitin enrichment after comparison with all metagenomic conditions. The presence of characterized enzymes related to the first step of chitin degradation in CAZy is given for each genus (indicated by plus ans minus signs for each enzyme AA10, CE4, GH18 and GH19). Bibliographical references reporting activity, or involvement of representative species, toward degradation of complex glycoside polymers is given (e.g. chitin and cellulose as well, as some of the characterized chitinase in CAZy were reported to have over-lapping activity toward cellulose). The chitin concentration where the genus abundance was found to be higher than controls is also given ([1x], [10x] or for both). Figure 6 in the original article is summarizing this table.
