# Supplementary material for: Soil Bacterial Community Shifts after Chitin Enrichment: An Integrative Metagenomic Approach
Source: PLoS One. 2013 Nov 20;8(11):e79699. doi: 10.1371/journal.pone.0079699 (PMC3835784; doi:10.1371/journal.pone.0079699)
Supplement: File S1 — Flowchart of the study. (DOCX) [file pone.0079699.s003.docx]

**File S1: Description of the pipeline used in this study**

This section is describing the global strategy applied in this work. When all the samples were gathered from the microcosm experiment (1), chitinase activity was measured from soil aqueous extracts for each biological replicates separately and also on the pool. (2) Metagenomic DNA has been extracted from each replicate separately, and stored until further analysis. As all of preliminary investigations rely on PCR (16S rRNA qPCR, RISA and phylochips), diluted metagenomic DNA aliquots were used. This allowed testing and optimizing of several parameters, including DNA sample pooling prior to amplification when high reproducibility could be statistically supported with the 3 replicates. (3) Because the chitinase activity assay is encompassing both Eukaryote and Prokaryote enzymes, we decided to assess the quantity of 16S rRNA gene copies in our metagenomic DNA samples through qPCR as a complement to detect how the bacterial community was reacting to incubation and enrichment. Each biological replicate has been tested separately in this experiment, and normalized according to DNA extraction yields. (4) Before undertaking deeper analyses, we assessed the changes in the bacterial community with RISA fingerprints (Ribosomal Intergenic Spacer Analysis). A strong reproducibility between RISA profiles from each replicate was highlighted (File S2). In a second step, we decided to pool the diluted DNA solution prior to amplification of the intergenic regions in order to see how this would impact or interpretation of results. The profiles we obtained were still coherent with what we previously got with replicates, thus, indicating that pooling didn’t modify the final interpretation of our fingerprints (File S2). (5) The phylochip technology has been selected in order to investigate at a deeper level the taxonomical changes we detected from preliminary analysis. In a first attempt, we tested all the replicates samples from day 20 (respectively: 0x20a, 0x20b, 0x20c, 1x20a, 1x20b, 1x20c, 10x20a, 10x20b, 10x20c) and the control soil at day 0 before incubation (0x0a, 0x0b, 0x0c) in order to verify the reproducibility. Again, the results we obtained showed high reproducibility between the replicates in terms of taxonomical profile at the genus level (File S3).

As only one time point could be investigated through shotgun pyrosequencing, a choice was made between samples from day 10 (endochitinase activity peak) and day 20 (after endochitinase activity peak). A new phylochip with pooled replicates prior to amplification was done in order to test, in a single run, all the 7 pooled samples of interest including day 10 (pooled 0x10, pooled 1x10, pooled 10x10), day 20 (pooled 0x20, pooled 1x20, pooled 10x20) and control 0x at day 0 (pooled 0x0). After analysis (File S3), more relevant taxonomical changes were identified at day 20, especially for sample 10x20, while the modifications at day 10 where not so pronounced for 10x10. Therefore, the samples from day 20 were selected for deeper analysis through shotgun pyrosequencing. (6) After careful consideration, only 2 of our 3 available replicates were used at day 20 through pyrosequencing. We sent the original stored metagenomic DNA solutions, as explained above in point (2). 6 samples were sent to pyrosequencing (respectively: 0x20a, 0x20b, 1x20a, 1x20b, 10x20a, 10x20b). The choice was made according to the preliminary information gathered from these samples, and only the 2 most similar amongst the 3 were selected. (7) In order to reinforce the comparison analysis, we add all the metagenomic datasets available on this soil. A “multi-level” analysis has been applied for metagenomic data interpretation, in order to clearly distinguish changes attributed to microcosm incubation and modifications due to chitin enrichment and concentration (File S4, Table S1, Table S2).
