# Supplementary material for: Soil Bacterial Community Shifts after Chitin Enrichment: An Integrative Metagenomic Approach
Source: PLoS One. 2013 Nov 20;8(11):e79699. doi: 10.1371/journal.pone.0079699 (PMC3835784; doi:10.1371/journal.pone.0079699)
Supplement: File S2 — RISA and description. (DOCX) [file pone.0079699.s004.docx]

**File S2: RISA profiles**


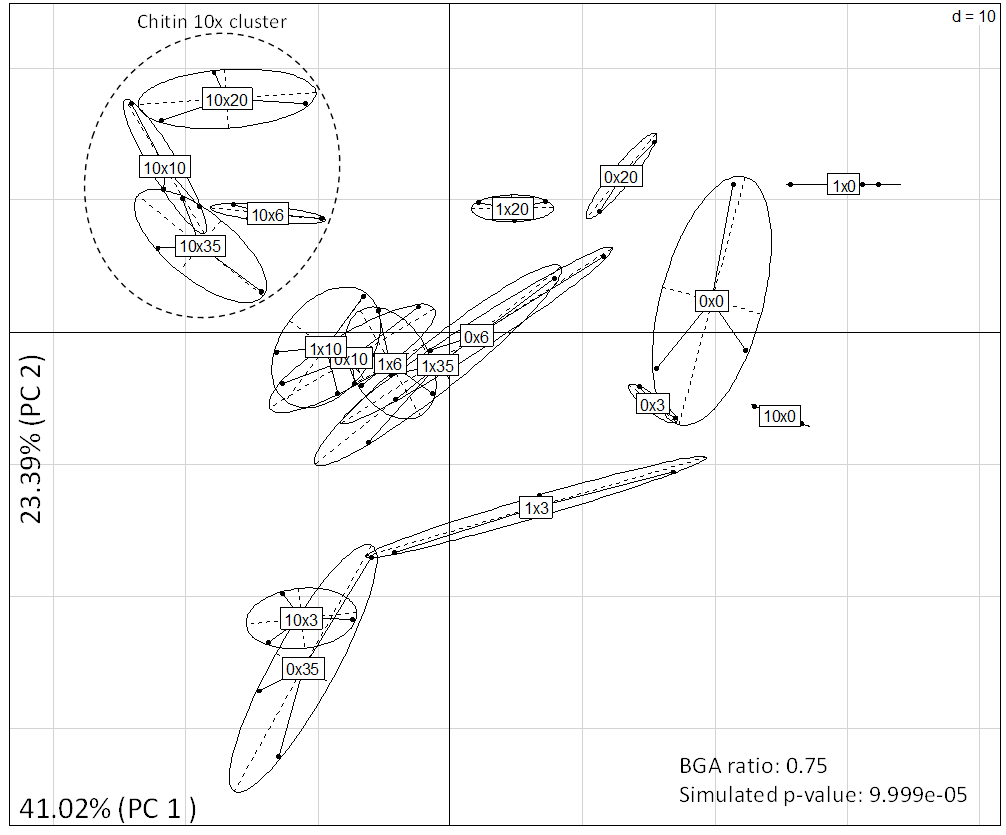


S2-Fig.1: Between Group Analysis (BGA) of the RISA profiles obtained on the metagenomic DNA of the 3 replicates for each condition. Each ellipse is representing the 3 bottle replicates for a given condition. BGA ratio indicates the distance modification from the former Principal Component Analysis after grouping the replicates together (1 = no modifications). The relevance of our grouping factor has been tested with a Monte Carlo simulation (n=10000), and the resulting simulated p-value is giving the probability to find a better BGA ratio than the one obtained by grouping the replicates together. Principal Component 1 and 2 are respectively displaying 41.02% and 23.39% of the variability observed in the RISA band profiles. Samples corresponding to high chitin treatment 10x are clearly clustering apart from the others (dotted ellipse).

S2-Fig.2: Reconstituted gel picture of RISA electropherograms profiles after pooling of the replicates. Each column represents the gel migration of amplified ribosomal intergenetic spacer sequences from each enrichment condition and time. The picture is flanked on both sides by ladders representing DNA fragments with known size, expressed in base pair. Numbers and arrows refer to specific comments in the text.

**Material and methods.** Whole bacterial community structure was assessed using Ribosomal Intergenetic Spacer Analysis (RISA). The intergenetic spacer (IGS) between the small (16S) and the large (23S) subunit of ribosomal sequences were amplified by PCR using primers 5'-TGCGGCTGGATCCCCTCCTT-3' (forward) and 5'-CCGGGTTTCCCCATTCGG-3' (reverse) [38]. 2µl of diluted metagenomic DNA (≈15ng) was mixed with 1.25µl of reverse and forward primers (10µM), 1µl Taq Polymerase (Invitrogen) and 20.5 µl of distilled sterile water. PCR was performed in a Biometra thermocycler with the following protocol: 95°C for 10mins and then 30 cycles of 95°C for 30s, annealing at 55°C for 30s, and elongation at 72°C for 1min, followed by 72°C for 15mins. 1µl PCR product was loaded on a chip into an Agilent DNA 7500 Lab, and electropherograms were performed and analyzed on an Agilent 2100 Bioanalyzer. Electropherograms were analyzed using principal component analysis (PCA) and between group analysis (BGA), with Rgui software 47. As a high reproducibility between replicates was identified (S2-Fig.1), new RISA profiles were done from pooled metagenomics DNA prior to amplification. Reconstituted RISA gels obtained after pooling the replicates are presented in S2-Fig.2.

**RISA profile analysis.** Complementary to the quantitative analysis (16S rRNA qPCR), a direct qualitative comparison between samples was carried out by analysis of RISA patterns obtained with PCR amplified ribosomal intergenic region. The preliminary analysis revealed high reproducibility between replicates (S2-Fig.1), indicating that the structure of the bacterial community is similar. Independently of the microcosm effect and regardless of the enrichment conditions tested, electropherograms show a strong similarity level all over the experimentation time indicating that the global structure of the bacterial community was preserved (S2-Fig.2). These preliminary analyses also revealed modifications of the structure overtime, which was explained by appearance of specific bands due to incubation in all the profiles (S2-Fig.2, arrow 1). This band even seems to be reinforced overtime, and also with chitin concentration, especially at day 6 (S2-Fig.2, arrow 2). However, some bands appearing at day 6 and maintained during the 29 remaining days can be specifically attributed to chitin treatments when compared with the control (S2-Fig.2, arrows 3). The community profiles obtained from high chitin concentration are rapidly clustering apart from the others, and seemed to stabilized between day 6 and 35 (S2-Fig.1), while the low chitin profiles are closely related to the controls.
