# Supplementary material for: Soil Bacterial Community Shifts after Chitin Enrichment: An Integrative Metagenomic Approach
Source: PLoS One. 2013 Nov 20;8(11):e79699. doi: 10.1371/journal.pone.0079699 (PMC3835784; doi:10.1371/journal.pone.0079699)
Supplement: File S3 — Phylochips and description. (DOCX) [file pone.0079699.s005.docx]

**File S3: Phylochip taxonomical profiles**

Preliminary phylochip experiments were performed on replicate samples from day 20 (0x20a, 0x20b, 0x20c, 1x20a, 1x20b, 1x20c, 10x20a, 10x20b, 10x20c) and with the original soil control from day 0 (0x0a, 0x0b, 0x0c) in order to test the reproducibility of the taxonomical profiles so obtained. Two arrays were hybridized with the 12 samples, and the taxonomical profiles were analyzed through Principal Component Analysis (PCA) and Between Group Analysis (BGA) with the Rgui software [48]. The results are presented in S3-Fig.1. The taxonomical profiles obtained at the genus level are displaying specific patterns strongly correlated to each biological condition, with a strong effect of incubation and high chitin treatment 10x (S3-Fig.1). Even if intra-variability was observed among the replicates (e.g. Incubation control 0x20, S3-Fig.1), each condition is clearly clustering apart from the others. However, the profiles obtained for the low chitin treatment 1x20 are displaying similarities with the untreated former soil 0x0. Taxonomical profiles of the highest chitin concentration are displaying higher diversity for *γ-proteobacteria*, and higher signal for *β-proteobacteria* (e.g. mostly due to *Burkholderia* genus).

S3-Fig.1: Principal Component Analysis (PCA) of the taxonomical profiles obtained from the preliminary phylochip at the genus level. Ellipses are representing the biological replicates of each condition after Between Group Analysis. BGA ratio indicates the distance modification from the former Principal Component Analysis after grouping the replicates together (1 = no modifications). The relevance of our grouping factor has been tested with a Monte Carlo simulation (n=10000), and the simulated p-value is giving the probability to find a better BGA ratio than the one obtained by grouping the replicates together. Principal Component 1 and 2 are respectively displaying 74.42% and 21.28% of the variability observed among the taxonomical profiles. Dotted lines are representing the vectors corresponding to specific prokaryote groups involved in the PCA.

The S3-Fig.2 is representing hybridization results obtained from the final phylochip, after pooling the replicates before amplification. The cluster analysis revealed a clear separation between control samples, showing lower diversity, and the chitin enriched samples, with higher diversity. Interestingly, the taxonomical profile of the low chitin treatment after 10 days 1x10 is clustering with the high concentration at day 20 10x20, while the low concentration at day 20 1x20 seems to be closer to the high concentration at day 10 10x10. However, the sample 10x20 was showing the greatest differences, and also the highest value of detected diversity. After careful consideration, we decided to sequence the samples from day 20, and show only the phylochip data from this time point in our work.

S3-Fig.2 Relative abundance of major bacterial groups detected on pooled phylochips. Stacked columns represent the percentage of total fluorescence signal detected for each prokaryote group, based on probe hybridizations. The number of identified genera per condition (n) is indicated in white at the column basis. Clusters were calculated on the taxonomical profiles at the genus level and exposed to bootstrap simulation (n=10000), with p-value at each nod. The grouping was done with Ward’s method based on variance analysis and distances are calculated according a correlation algorithm (the complement 1*-r* of Pearson's *r* correlation). Dendrogram scale is given the similarity based on the Euclidean distance.
