# Supplementary material for: Soil Bacterial Community Shifts after Chitin Enrichment: An Integrative Metagenomic Approach
Source: PLoS One. 2013 Nov 20;8(11):e79699. doi: 10.1371/journal.pone.0079699 (PMC3835784; doi:10.1371/journal.pone.0079699)
Supplement: File S4 — Metagenomic analysis and description. (DOCX) [file pone.0079699.s006.docx]

**File S4: Multistep analysis of taxonomical and functional metagenomic annotations**

**Metagenomic analysis at *sensu stricto* with 0x20 incubation control.** The only relevant control for detecting the chitin impact was the metagenomic condition 0x20 corresponding to the soil incubated in the same conditions as the chitin treated 10x20. Analysis of the metagenome sequences indicated that the relative proportion of *Actinobacteria*, *γ-proteobacteria* and *β-proteobacteria* was found to be increased in 10x20, while *Planctomycetes*, *Cyanobacteria*, *α-* and *δ-proteobacteria* were reduced (S4-Fig.1). Unfortunately, the changes in the community could not be statistically supported every time because of the low repetition number. Cross comparison revealed 35 genera mostly related to *γ-proteobacteria* and *Actinobacteria*, as well as 91 genera significantly enriched respectively in 10x20 metagenomes and incubation control 0x20 (S4-Fig.1). At this level, these observations rely on changes in the relative distribution of sequences from quantitatively constant DNA solutions and cannot discriminate between a negative biological effect of chitin on some genera or a simple mathematic compensation after the growth of the 35 genera positively affected by chitin. Similar trends were also detected at the functional level with 412 and 244 SEED functions widely distributed in all subsystems whose detection was increased in 10x20 and 0x20 respectively (S4-Fig.2).

**Metagenomic analysis at *sensu medio* with 0x0 untreated control.** When analyzing metagenomes by including the non-incubated control soil (0x0, *sensu medio*), the so-called microcosm effect is strongly highlighted. The strong increase of *Actinobacteria* and at a lesser extent of *α-proteobacteria* in incubated control 0x20 in comparison to 0x0 led to a proportional decrease of most of the other phyla (Fig.4, original article). This was also noticed at lower taxonomical level, with a selection of 91 genera mostly related to *Actinobacteria* and *α-proteobacteria* in 0x20 (S4-Fig.1). The same trend was also detected at the functional level, with a clear effect of incubation with 458 and 260 functions whose occurrence was decreased and increased in 0x20, respectively (S4-Fig.2). However, addition of this control condition 0x0 to the initial one 0x20 increased the estimated impact of chitin on the community structure with 71 and 182 genera whose occurrence was found to be increased and decreased in 10x20 (S4-Fig.1). Similarly at the functional level, 430 and 418 functions were respectively augmented and reduced in 10x20 (S4-Fig.2). In addition, cross-comparison between the two controls 0x20 and 0x0 helps in removing the microcosm effect to focus only on genera and functions impacted by chitin. Overall, 48.6% (88/181) and 70.4% (50/71) of the genera, as well as 30.7% (132/430) and 38.3% (160/418) of the functions were already impacted at *sensu stricto* by incubation, while the remaining part can specifically be attributed to chitin treatment (S4-Fig.2).

**Metagenomic analysis at *sensu lato* with Rothamsted database.** In a new step, a *sensu lato* statistical analysis was carried out by integrating the 4.88 x 10^9^ bp of metagenomic sequence data already available for this soil as a reference control 33. The wide range of sampling and methodological conditions included in this database will reinforced the analysis in order to specifically identify taxa and functions affected by chitin amendment. Addition of the 11 metagenomes of the Rothamsted database significantly increases the robustness of the statistical analysis (n=11), strengthening some relevant changes and invalidating other ones. For instance, some of the previous observation realized at *sensu stricto* and *medio* could not be supported anymore at this level. This is the case for the increase of *α-proteobacteria*, leaving the *Actinobacteria* phylum to be the only one whose representative occurrence was increased after chitin treatment (Fig.4, original article). The number of positively affected genera (35, 71 and 94 at respectively, *sensu stricto, medio* and *lato* analytical levels) is revealing a wider range of putative chitin degraders masked by the lack of analysis stringency (S4-Fig.1). The number of bacteria phyla whose occurrence was decreased by the strongest chitin amendments (16 phyla, S4-Tab.1) was significantly reduced in comparison with the previous *sensu stricto* and *sensu medio* analysis, including *Proteobacteria* through *δ-* and *ε-proteobacteria* classes, as well as *Bacteroidetes, Chlorobi, Spirochaetes, Aquificae, Fusobacteria, Fibrobacteres, Elusimicrobia, Deferribacteres, Tenericutes, Chrysiogenetes, Candidatus Poribacteria, Nanoarchaeota and Chrysiogenetes*. In addition, this step by step analytical procedure contributed to clearly dissociate the microcosm incubation effect from the impact of chitin enrichment by specifically identifying the taxa already positively (63 out of 94 genera, 67%) and negatively (55 out of 116 genera, 47.7%) influenced by incubation (S4-Fig.1). The increase of analytical stringency related to the Rothamsted database also decreased the number of functions whose occurrence was found to be negatively affected by microcosm incubation and chitin amendment (respectively, 361 in comparison to 458 and 343 in comparison to 418 at *sensu medio*, S4-Fig.2). Interestingly, the number of functions whose occurrence is scaled up by microcosm incubation (808) and chitin amendment (1138) is increased at *sensu lato* in comparison to previous ones (*sensu stricto* and *sensu medio*). Again, the chitin effect can easily be distinguished considering that the microcosm effect was responsible for only 28.9% (329/1138) and 35% (120/343) of the total number of functions that were respectively increased and decreased (S4-Fig.2).


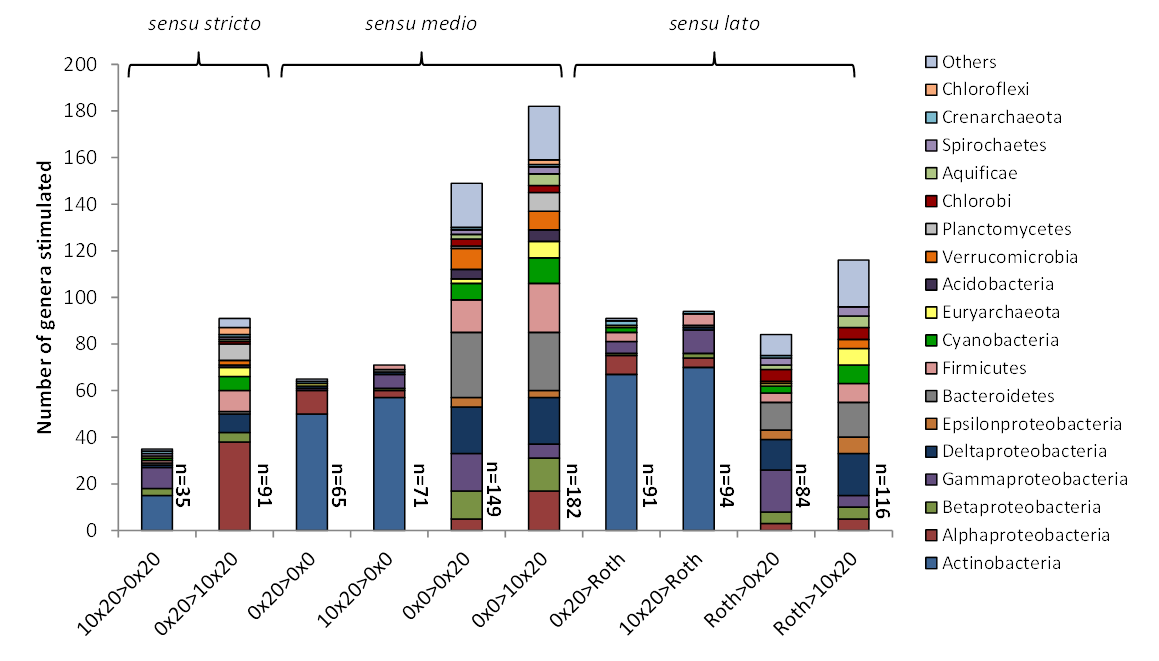


S4-Fig.1: Cross-comparison of the taxonomic profiles of all metagenomic conditions. Analysis has been performed on metagenomes at the genus level through ANOVA (p-value cut-off < 0.05). The panel displays the cross-comparison of all conditions, and indicates the number of genera (n) significantly more abundant in metagenomes of one condition against another. The conditions are indicated as follow: The 11 metagenomes from Rothamsted Roth, the 2 controls before incubation 0x0, the 2 incubation controls 0x20, and chitin treated metagenomes 10x20. The comparison with 1x20 is not presented as it is very similar to the control 0x20. The comparison between 0x0 and Rothamsted is also not presented for the same reasons.


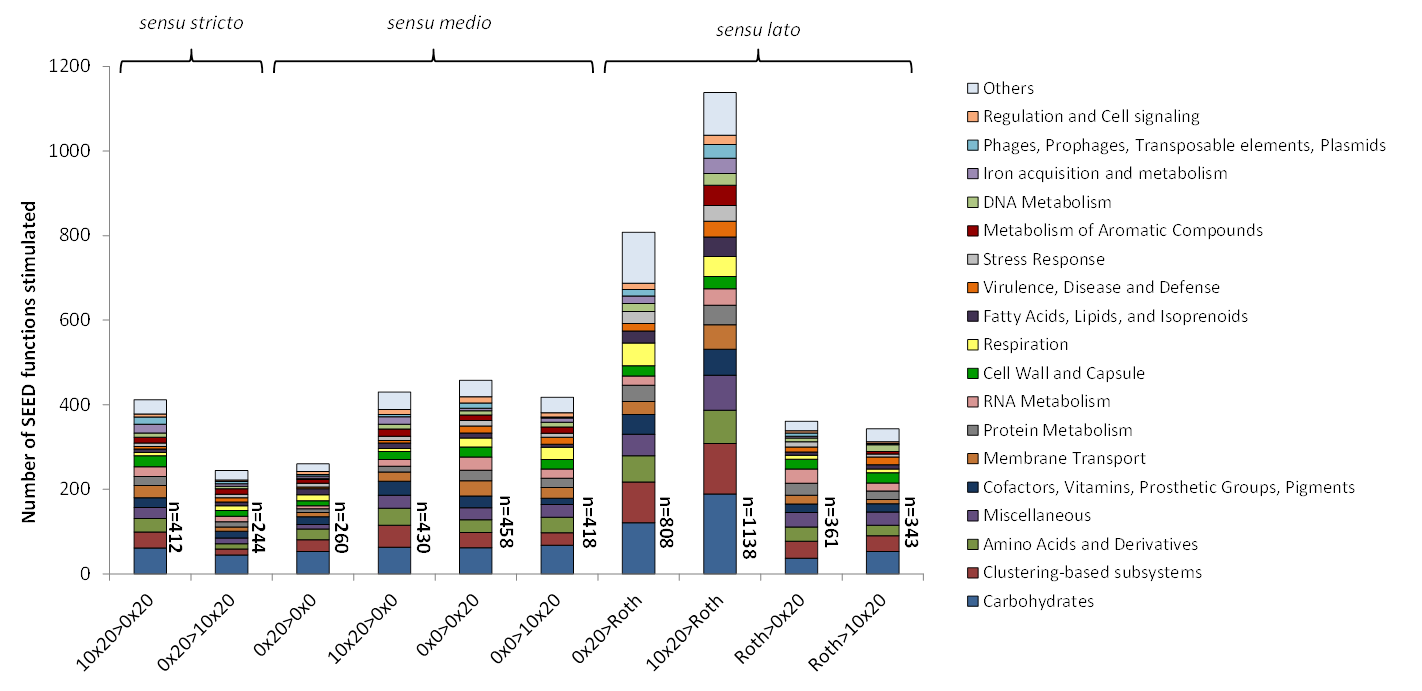


S4-Fig.2: Cross-comparison of the functional profiles of all metagenomic conditions. Analysis has been performed on metagenomes at the SEED functional subsystem level through ANOVA (p-value cut-off < 0.05). The panel displays the cross-comparison of all conditions, and indicates the number of functions (n) significantly more abundant in metagenomes of one condition against another. The conditions are indicated as follow: The 11 metagenomes from Rothamsted Roth, the 2 controls before incubation 0x0, the 2 incubation controls 0x20, and chitin treated metagenomes 10x20. The comparison with 1x20 is not presented as it is very similar to the control 0x20. The comparison between 0x0 and Rothamsted is also not presented for the same reasons.
